# Acute modulation of synaptic plasticity of pyramidal neurons by activin in adult hippocampus

**DOI:** 10.3389/fncir.2014.00056

**Published:** 2014-06-02

**Authors:** Yoshitaka Hasegawa, Hideo Mukai, Makoto Asashima, Yasushi Hojo, Muneki Ikeda, Yoshimasa Komatsuzaki, Yuuki Ooishi, Suguru Kawato

**Affiliations:** ^1^Department of Biophysics and Life Sciences, Graduate School of Arts and Sciences, The University of TokyoMeguro, Japan; ^2^Bioinformatics Project (BIRD), Japan Science and Technology Agency, The University of TokyoMeguro, Japan; ^3^Core Research for Evolutional Science and Technology Project of Japan Science and Technology Agency, The University of TokyoMeguro, Japan; ^4^Department of Computer Science, School of Science and Technology, Meiji UniversityKawasaki, Japan; ^5^National MEXT Project in Special Coordinate Funds for Promoting Science and Technology, The University of TokyoMeguro, Japan

**Keywords:** hippocampus, activin, spine, LTP, rapid effect, synapse, kinase

## Abstract

Activin A is known as a neuroprotective factor produced upon acute excitotoxic injury of the hippocampus (in pathological states). We attempt to reveal the role of activin as a neuromodulator in the adult male hippocampus under physiological conditions (in healthy states), which remains largely unknown. We showed endogenous/basal expression of activin in the hippocampal neurons. Localization of activin receptors in dendritic spines (=postsynapses) was demonstrated by immunoelectron microscopy. The incubation of hippocampal acute slices with activin A (10 ng/mL, 0.4 nM) for 2 h altered the density and morphology of spines in CA1 pyramidal neurons. The total spine density increased by 1.2-fold upon activin treatments. Activin selectively increased the density of large-head spines, without affecting middle-head and small-head spines. Blocking Erk/MAPK, PKA, or PKC prevented the activin-induced spinogenesis by reducing the density of large-head spines, independent of Smad-induced gene transcription which usually takes more than several hours. Incubation of acute slices with activin for 2 h induced the moderate early long-term potentiation (moderate LTP) upon weak theta burst stimuli. This moderate LTP induction was blocked by follistatin, MAPK inhibitor (PD98059) and inhibitor of NR2B subunit of NMDA receptors (Ro25-6981). It should be noted that the weak theta burst stimuli alone cannot induce moderate LTP. These results suggest that MAPK-induced phosphorylation of NMDA receptors (including NR2B) may play an important role for activin-induced moderate LTP. Taken together, the current results reveal interesting physiological roles of endogenous activin as a rapid synaptic modulator in the adult hippocampus.

## Introduction

Activin A is a homodimer of inhibin β_A_ polypeptides, which belongs to the superfamily of transforming growth factor-β (TGF-β) (Pangas and Woodruff, 2000). In addition to its roles in development (Asashima et al., 1990; Kokan-Moore et al., 1991) and hormonal regulation via hypothalamic-pituitary-gonadal (HPG) axis (Ling et al., 1986; Vale et al., 1986; Gregory and Kaiser, 2004), recent studies show new important functions of activin in tissue repair, fibrosis and inflammatory disease in various organs, including the brain (Hughes et al., 1999; Wu et al., 1999). Activin binds to type II receptor (Ser/Thr kinase receptor) and dimerization of type II and type I receptors occurs, resulting in phosphorylation the Smad family transcription factors, which then leads to gene expression (Pangas and Woodruff, 2000; Derynck and Zhang, 2003). Smad-independent pathways are also activated by activin receptors, including mitogen-activated protein kinase (MAPK) signaling (Ten Dijke et al., 2000; Derynck and Zhang, 2003; Werner and Alzheimer, 2006).

Recent advances shed light on neuroprotective actions of activin in the hippocampus, a center of learning and memory, during excitotoxic injury. For example, activin is essential in the neuroprotective action following acute excitotoxic lesion of the hippocampus (Tretter et al., 1996, 2000). Application of recombinant activin shows neuroprotective effects in cultured hippocampal neurons upon toxic treatments (Tretter et al., 1996; Iwahori et al., 1997). A significant elevation of the expression of mRNA of activin is demonstrated in the dentate gyrus (DG) of the hippocampus upon excitotoxic high frequency stimulation (Inokuchi et al., 1996) or after acute injury (Lai et al., 1996, 1997; Tretter et al., 1996, 2000).

Activin A can also modulates synaptic plasticity of normal adult hippocampus under some physiological conditions. Activin plays a role in maintaining the late long-term potentiation (late LTP, 24 h after stimulation) in adult hippocampal DG and long-term memory *in vivo* (Ageta et al., 2010). Activin increases neck length of spines (morphological change) as well as increasing synaptic contacts in primary cultured neurons, although the head size and the density of spines are not affected (Shoji-Kasai et al., 2007).

To examine deeply the essential functions of activin in adult neural circuit of hippocampus under physiological conditions, we investigated the rapid effect (1~2 h) of activin including spinogenesis and early LTP in the hippocampus of adult male rat. To reveal molecular mechanisms of spinogenesis in downstream of activin receptors, we focus on the role of several kinases which are essential for synaptic plasticity. We also performed identification of the cellular localization of activin as well as subcellular localization of activin type IB receptors in the hippocampus, which had been unknown.

## Materials and methods

### Animals

Young adult male Wistar rats (12 week-old, 280–320 g) were purchased from Saitama Experimental Animals Supply (Japan) and Harlan Sprague Dawley (Indianapolis, IN). All animals were maintained under a 12 h light/12 h dark cycle and free access to food and water. The experimental procedure of this research was approved by the Committee for Animal Research of the University of Tokyo.

### Chemicals

Lucifer Yellow was obtained from Molecular Probes (USA). Cyano-nitroquinoxaline-dione (CNQX), MK-801, PD98059, SB203580, LY294002, cyclosporin A (CsA), cycloheximide (CHX), actinomycin D (actD), Ro25-6981 and N-methyl-D-aspartate (NMDA) were purchased from Sigma (USA). H-89 and KN-93 were from Calbiochem (USA). Recombinant activin A and follistatin were kind gifts from Ajinomoto Co. (Japan).

### Immunohistochemical staining of hippocampal slices

Immunohistochemical staining was performed essentially as described in previous references (Kimoto et al., 2001; Kawato et al., 2002; Hojo et al., 2004). Briefly, hippocampal slices were prepared from rat deeply anesthetized and perfused transcardially with phosphate-buffered saline [PBS; 0.1 M phosphate buffer and 0.14 M NaCl (pH 7.3)], followed by fixative solution of 4% paraformaldehyde. The hippocampi were postfixed, cryoprotected and frozen-sliced coronally at 20 μm thickness with a cryostat (Leica CM1510, Germany). Brains from 3 animals were used, and from each brain two representative coronal sections including the dorsal hippocampus was selected.

To investigate the distribution of activin type IB receptor and activin A in the hippocampal formation, we used anti-activin receptor antiserum (TAL-8043) and anti-activin A antiserum (TT122G), respectively. These antisera were successfully used in former studies (Koyano et al., 2002; Fukui et al., 2003).

After application of antiserum at 1/500 dilution, the hippocampal slices were incubated at 4°C for 18 h in the presence of 0.5% Triton X-100 and 3% skim milk with gentle shaking. Biotinylated anti-rabbit IgG (1/1000) in PBS was then applied, followed by a 30 min incubation with streptavidin-horseradish peroxidase complex (Vector Laboratories, USA). Immunoreactive cells were detected in diaminobenzidine-nickel. After dehydration and embedding in Entellan Neu (Merck), the immunoreactive cells were examined under microscope, and digital images with a 2272 × 1704 pixel resolution were taken by a digital camera (COOLPIX4500, Nikon).

### Postembedding immunogold method for electron microscopy

Hippocampal slices were prepared by slicing at 4°C using a vibratome (Leica, Germany). Freeze substitution and low-temperature embedding of the specimens was performed as described elsewhere (Mukai et al., 2007). Briefly, slices were plunged into liquid propane in a Cryofixation System KF80 (Reichert-Jung, Austria). The samples were immersed in uranyl acetate solution in a cryosubstitution AFS unit (Leica, Austria), and infiltrated with Lowicryl HM20 resin. After polymerization, ultrathin sections (80 nm thickness) were cut using a Reichert-Jung ultramicrotome and mounted on mesh grids. For immunolabeling, sections were incubated with primary antibody for activin receptor (1/5000) overnight, and incubated with secondary gold-tagged (10 nm) Fab fragment in TBS. Sections were counterstained with 1% uranyl acetate, and viewed on a JEOL 1200EX electron microscope (Japan). A search for immunogold-labelings was performed for at least 30 synapses per each rat.

### Polymerase chain reaction

The PCR reaction mixture contained [cDNA (corresponding to 50 ng of total RNA), 1 X GC buffer I, 0.2 mM dNTP mixture, 0.2 μ M of each primer, 1.25 units of TaKaRa LA Taq (TaKaRa Bio, Japan)] in the total volume of 25 μ L. PCR cycle condition is as follows : (1) 95°C denaturing step for 30 s, (2) 58–65°C annealing step for 20 s and (3) 72°C elongation step for 30 sc with first 95°C denaturing for 1 min and last elongation for 15 min. To determine the optimal number of cycles for samples from hippocampi, various number of cycles were performed during PCR amplification. The primers used and amplification conditions were shown in Table 1. PCR products (5 μ l) were electrophoresed on 2.0% agarose gels. Gels were stained with ethidium bromide, and visualized under UV light. Images were recorded with Printgraph (ATTO, Japan). For quantitative analysis, images of the bands were analyzed using NIH Image software.

**Table 1:** **Primers used for RT-PCR analysis of gene expression, and PCR experimental condition**.

**Gene**	**Direction**	**Primer sequence : (5′-3′)**	**Product length (bp^a^)**	***T*^b^_a_(°C)**	**Cycles**
Inhibin α	Forward	TGTCGTCAGGGCAAGAGAACTATG	401	58	31
	Reverse	ACCTGGTGGCTGCGTATGTGT			
Inhibin β_A_	Forward	GGGTAAAGTGGGGGAAAACGGGTATG	412	65	31
	Reverse	GCGCTGGATGCTGCTAGACACTGG			
Inhibin β_B_	Forward	GGCCGGCCCAACATCACG	393	63	31
	Reverse	GTCCACCTTCTTCTCCACCACATTCC			
Inhibin β_C_	Forward	CTCAGCCAGCGCCCCATACTCA	221	63	31
	Reverse	TGCAGGACCTCCACACCACCAGTAG			
Inhibin β_E_	Forward	CTGACACCCCAAGGAGAACG	431	65	31
	Reverse	CCGCTAGAGGGCAGAGTCAG			
GAPDH	Forward	TATGACTCTACCCACGGCAAGTTCAA	830	60	21
	Reverse	ACCACCCTGTTGCTGTAGCCATATTCAT			

a bp, base pair(s);

b T_a_, annealing temperature.

Nucleotide sequences referred were as follows; BC08564 and M36453 for inhibin α subunit, M37482 for inhibin β_A_ subunit, M32756-M32758 for inhibin β_B_ subunit, AF140031 for inhibin β_C_ subunit, AF089825 and AF140032 for inhibin β_E_ subunit. Each pair of PCR primers was designed on different exons of the target gene to avoid amplification of genomic DNA.

### Sequencing

PCR products on the electrophoresed agarose gels were extracted using QIAquick gel extraction kit (QIAGEN, Germany) and cloned into pGEM-T-Easy vector (Promega, USA). Dye terminator cycle sequencing was performed using Thermosequenase II sequencing kit (Amersham Biosciences, USA), and sequenced by ABI373A DNA sequencer (Applied Biosystems, USA).

### Slice preparation for spine analysis

Adult male rats were deeply anaesthetized and decapitated. Immediately after decapitation, the brain was removed from the skull and placed in ice-cold oxygenated (95% O_2_, 5% CO_2_) artificial cerebrospinal fluid (ACSF) containing (in mM): 124 NaCl, 5 KCl, 1.25 NaH_2_PO_4_, 2 MgSO_4_, 2 CaCl_2_, 22 NaHCO_3_, and 10 D-glucose (all from Wako); pH was set at 7.4. The hippocampus was then dissected and 300 μm thick transverse slices to the long axis, from the middle third of the hippocampus, were prepared with a vibratome (Dosaka, Japan). These slices were “fresh” slices without ACSF incubation. Slices were then incubated in oxygenated ACSF for 2 h (slice recovery processes) in order to obtain widely used “acute slices.”

### Imaging and analysis of dendritic spine morphology

#### Current injection of Lucifer Yellow

“Acute” slices were incubated with 10 ng/mL activin A or drugs including kinase inhibitors. Slices were then prefixed with 4% paraformaldehyde in PBS at 4°C for 2–4 h. Neurons within slices were visualized by an injection of Lucifer Yellow under Nikon E600FN microscope (Japan) equipped with C2400-79H infrared camera (Hamamatsu Photonics, Japan) and with 40× water immersion lens (Nikon). Dye injection was performed with glass electrode whose tip was filled with 5% Lucifer Yellow for 15 min, using Axopatch 200B (Axon Instruments, USA). Approximately 5 neurons within a 100–200 μm depth from the surface of a slice were injected (Duan et al., 2002).

#### Confocal laser microscopy and morphological analysis

The imaging was performed from sequential z-series scans with LSM5 PASCAL confocal microscope (Zeiss, Germany) at high zoom (3.0) with a 63× water immersion lens, NA 1.2 (Zeiss). For Lucifer Yellow, the excitation and emission wavelengths were 488 and 515 nm, respectively. For analysis of spines, three-dimensional image was reconstructed from approximately 40 sequential z-series sections of every 0.45 μm. The applied zoom factor (3.0) yielded 23 pixels per 1 μm. The confocal lateral (XY) resolution was approximately 0.26 μm. The head diameter is determined from examination of all XY planes with different z-stages which contain a target spine. The head diameter could be, therefore, determined even in the range of 0.2–0.5 μm. Confocal images were then deconvoluted using AutoDeblur software (MicroCybernetics, USA). The density of spines as well as the head diameter was analyzed with Spiso-3D (mathematical and automated software calculating geometrical parameters of spines) developed by Bioinformatics Project of Kawato's group (Mukai et al., 2011; Ooishi et al., 2012a). Spiso-3D has an equivalent capacity with Neurolucida (MicroBrightField, USA) which however needs time-consuming manual operation. We analyzed the secondary dendrites in the stratum radiatum, lying between 100 and 250 μm from the soma. The spine density was calculated from the number of spines having a total length of 50–80 μm. To distinguish different responses in spine subpopulations, spines were classified into three categories according to their head diameters: (1) A small-head spine, which has head diameter (D) between 0.2 and 0.4 μm, (2) A middle-head spine, which has head diameter between 0.4 and 0.5 μm. (3) A large-head spine, which has head diameter 0.5–1.0 μm. These three categories were useful to distinguish different responses upon kinase inhibitor application. Because the majority of spines (>95%) had a distinct head and neck, and stubby spines and filopodium did not contribute much to overall changes, we analyzed mainly spines having a distinct head.

## LTP measurements upon weak-theta burst stimulation (TBS) in CA1

The same “acute” slices used for spinogenesis experiments were used for LTP investigations. The acute slice was incubated with activin A at 10 ng/ml (0.4 nM) for another 2 h. The slice was then transferred to an interface recording chamber, continuously perfused (2 ml/min) with oxygenated ACSF at 32°C.

Experimental details with custom multielectrode probes are described elsewhere (Mukai et al., 2007). Briefly, slices were positioned on a custom multielectrode probe in which 64 planar microelectrodes (Alpha MED Scientific, Japan) were particularly designated to densely cover the important regions containing essential synaptic contacts of pyramidal neurons of the stratum radiatum in CA1. EPSP responses were measured with selected electrodes in CA1. We determined the input-output curve of fEPSP by gradually increasing the stimulus intensity. The interval of the stimulation was 45 s. When responses were saturated, we calculated the stimulus intensity which gave the half maximum of fEPSP.

For induction of small sub-threshold long-term potentiation (small LTP), “weak-TBS” was applied to the Schaffer collaterals. Here the weak-TBS stimuli were delivered as discrete 3 bursts separated by 200 ms (Kramar et al., 2009). One burst consists of 5 pulses at 100 Hz. On the other hand, “full-TBS,” consisting of total 50 pulses, was applied to obtain full LTP.

### Western immunoblot analysis

Purified hippocampal fractions prepared by centrifugation were suspended in 125 mM Tris-HCl buffer (pH 6.8), containing 5 mM 2-mercaptoethanol, 10% sucrose, 6% sodium dodecylsulfate and 0.002% bromophenol blue. The fractions were subjected to electrophoresis using a 10% polyacrylamide gel. After transfer to polyvinylidene fluoride membranes (Immobilon-P; Millipore, USA), the blots were probed with anti-activin receptor antiserum (TAL-8043, 1/10000) for 15 h at 4°C, and incubated with horseradish peroxidase (HRP) conjugated goat anti-rabbit IgG (Cell Signaling, USA). We also verified the specificity of anti-activin antiserum (TT122G, 1/3000) against purified activin. The protein bands were detected using ECL plus Western blotting detection reagents (Amersham, USA). To obtain high quality images of chemiluminescence from protein bands using ECL plus, we used LAS3000 Image Analyzer (Fuji Film) with a 16-bit wide dynamic range.

### Statistical analysis

For spine morphological experiments, the significance of activin or drug effect was first examined via One-Way ANOVA (analysis of variance) followed by Tukey-Kramer *post-hoc* multiple comparisons test. For LTP analysis, we defined the LTP ratio as the average of EPSP slope level for *t* = 50–60 min. Two-Way ANOVA was used for multiple comparisons between inhibitor treatments in LTP experiments. Significance was defined as ^*^p < 0.05, ^**^p < 0.01.

## Results

### Activin expression in the hippocampus under physiological conditions

**Figure 1:**
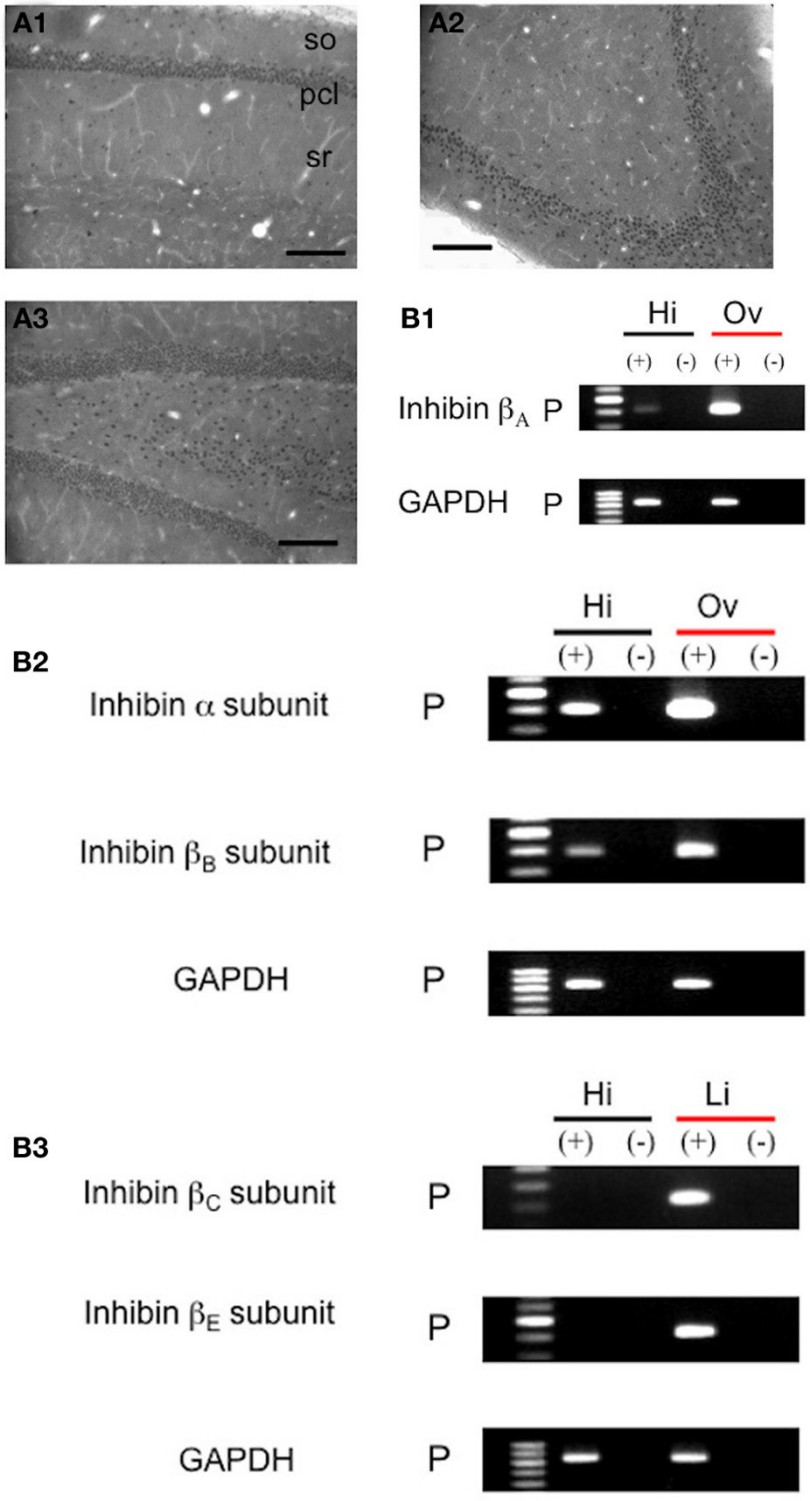
**Expression and localization of activin in the adult male rat hippocampus under physiological conditions**. **(A)** Immunohistochemical staining of activin with anti-activin IgG. **(A1)** Coronal section of CA1 region. **(A2)** Coronal section of CA3 region. **(A3)** Coronal section of DG. so, stratum oriens; pcl, pyramidal cell layer; sr, stratum radiatum. Scale bar, 200 μm. Representative images are shown from approx. 18 photographs from 6 independent slices from 3 animals. (Supplementary Figure 1A4) No staining after preadsorption treatments with activin. **(B)** RT-PCR analysis of mRNA for inhibin β_A_, α, β_B_, β_C_ and β_E_ subunits in the hippocampus (31 cycles). **(B1)** The expression of inhibin β_A_ subunit transcripts. From left to right, size marker (Marker), hippocampus (Hi), ovary (Ov). **(B2)** Inhibin α and β_B_ subunit. **(B3)** Inhibin β_C_ and β_E_ subunits. (+): with reverse transcriptase added; (−): without reverse transcriptase, a negative control. P: Ethidium bromide staining of PCR products. GAPDH (21 cycles) was used as an internal control for PCR amplification. Ovary (Ov) or Liver (Li) was used for positive control. The image is a representative one from duplicate determinations for each rat of total 4 rats.

We examined the distribution of endogenous activin by immunohistochemistry, and found the expression of activin molecules in the hippocampus in physiological state (Figure 1). Activin-immunoreactivity was associated in neurons of the CA1, CA3, and DG region in the hippocampal formation (Figure 1). With a resolution of light microscopy, only cell body expression is visible. Results of RT-PCR also supported the existence of mRNA of inhibin β_A_, subunit of activin molecule in the hippocampus (Figure 1, Table 1). Western blot analysis was performed to confirm the specificity of antisera used for immunohistochemistry (Figure S1).

### Molecular biological analysis of activin

The expression of inhibin β subunit transcripts, which make activin in the hippocampus, was confirmed via semi-quantitative RT-PCR. The relative level of inhibin β_A_ mRNA in the hippocampus was approx. 1/20 of that in the 12 week-old ovary (Figure 1). Other members of inhibin family were also expressed in the hippocampus (Figure 1). The resulting sequences were identical to the reported cDNA sequences of these molecules.

### Synaptic localization of activin receptor in the hippocampus

**Figure 2:**
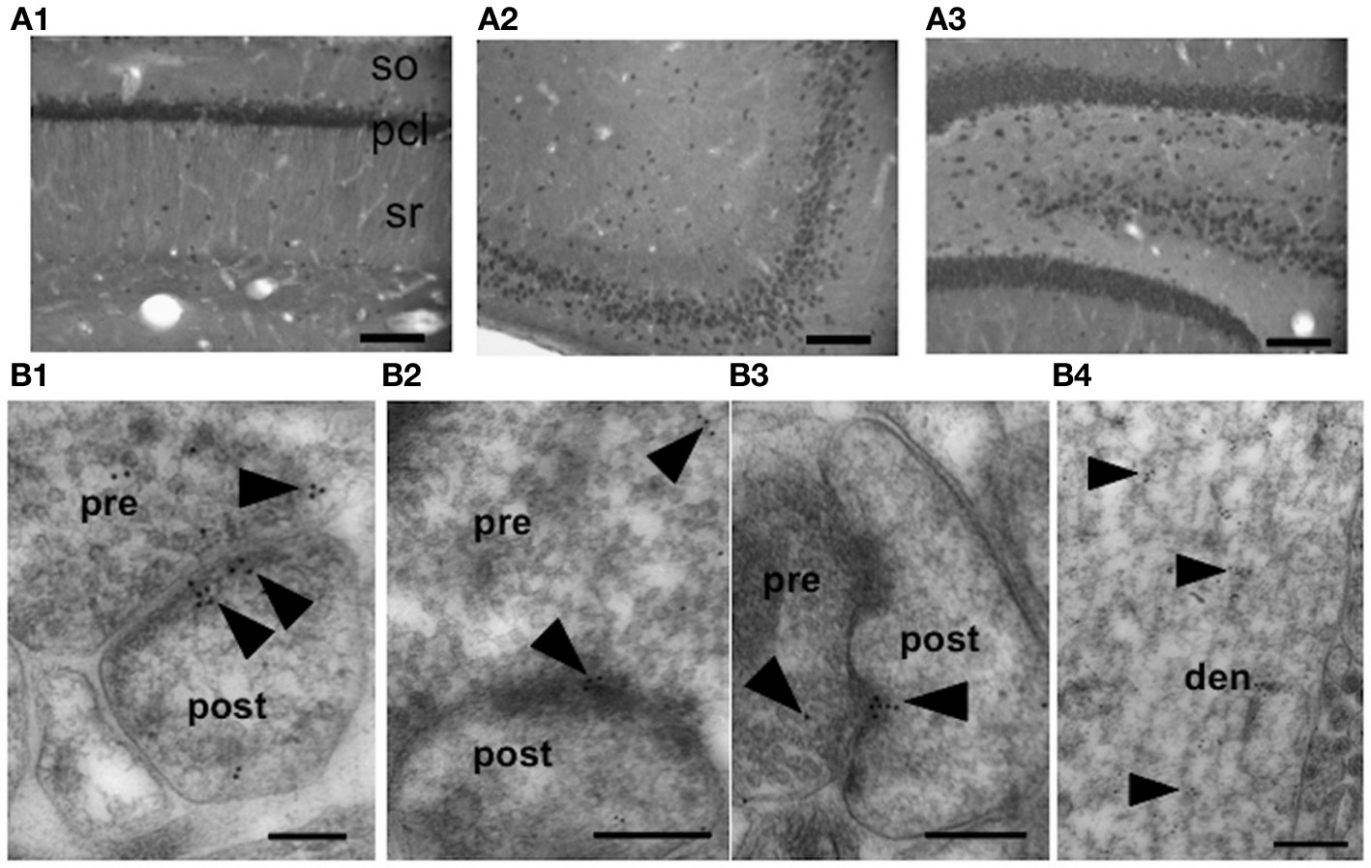
**Localization of activin type IB receptor in the rat hippocampus. (A)** Immunohistochemical staining of the hippocampal slice (coronal section) of adult male rat probed with anti-activin receptor IgG. **(A1)** CA1, **(A2)** CA3, **(A3)** DG. Scale bar, 200 μm. so, stratum oriens; pcl, pyramidal cell layer; sr, stratum radiatum. Representative images are shown from approx. 18 photographs from 6 independent slices from 3 animals. (Supplementary Figure 2A4) No staining after preadsorption treatments with activin receptor. **(B)** Immunoelectron microscopic analysis, using anti-activin receptor IgG, of the distribution of activin receptor within the axospinous synapses of the hippocampal principal neurons in the stratum radiatum of CA1 **(B1)**, stratum radiatum and lucidum of CA3 **(B2)**, and hilus of DG **(B3)**. Representative images are shown from approx. 100 photographs from 27 independent slices from 4 animals. Gold particles (arrowheads) were localized in the pre- and postsynaptic regions **(B1–3)**. In spines (postsynapses), gold particles were associated with PSD regions as well as within the spine head **(B1)**. In the presynaptic terminus, gold particles were often associated with small synaptic vesicles. In dendrites of neurons (**B4**, CA1 region), gold particles were often found in cytoplasmic space. A 1:20000 dilution anti-activin receptor IgG was used to prevent non-specific labeling. pre, presynaptic region; post, postsynaptic region; den, dendrite. Scale bar, 200 nm for **(B1,B3)**, 300 nm for **(B2,B4)**.

The cellular localization and expression of activin receptor protein has been unknown. First we tried to reveal light microscopic investigations of the immunohistochemical staining using anti-activin receptor antiserum (TAL-8043; 1/5000) were performed to determine the cellular localization of activin receptor in the hippocampal formation of adult male rats (Figure 2). Activin receptors distributed over the principal neurons in CA1, DG regions. With a resolution of light microscopy, only cell body expression is visible. CA3 neurons had less pronounced immunoreactivity. Only weak immunoreactivity was associated in glial cells. Western blot analysis using anti-activin receptor antiserum yielded the main band of 57 kDa activin receptor protein (type IB) present in the hippocampus (Figure S1).

Further, we identified subcellular distribution (particularly synaptic and dendritic localization) of activin receptor in hippocampal neurons using anti-activin receptor antiserum (TAL-8043; 1/5000). An immunoelectron microscopic analysis using post-embedded immunogold was performed, since this method can determine synaptic and dendritic localization of activin receptor with a molecular resolution. The activin receptor was observed in both the axon terminals and dendritic spines of principal neurons (Figure 2). Gold particles were clustered in the postsynaptic and presynaptic compartments, dendrites and cytoplasm. At postsynapses (spines), gold particles were found within the cytoplasm of the spine head. In some cases, gold particles were affiliated within the postsynaptic density. At the presynaptic terminals, gold particles were associated with synaptic vesicles. Significant labeling along dendrites was also found, however, much less gold particles were observed in axons. Multiple labeling (3 or more) of immunogold in the pre- and post-synaptic compartments was confirmed to ensure the specific labeling. A search for immunogold-labeled activin receptor proteins was performed for at least 30 synapses per each CA1, CA3 and DG region from more than 100 independent images. The topological distributions of gold particles within the neurons of the CA1, CA3 and DG were essentially identical.

### Activin promotes acute spinogenesis in the hippocampus

**Figure 3:**
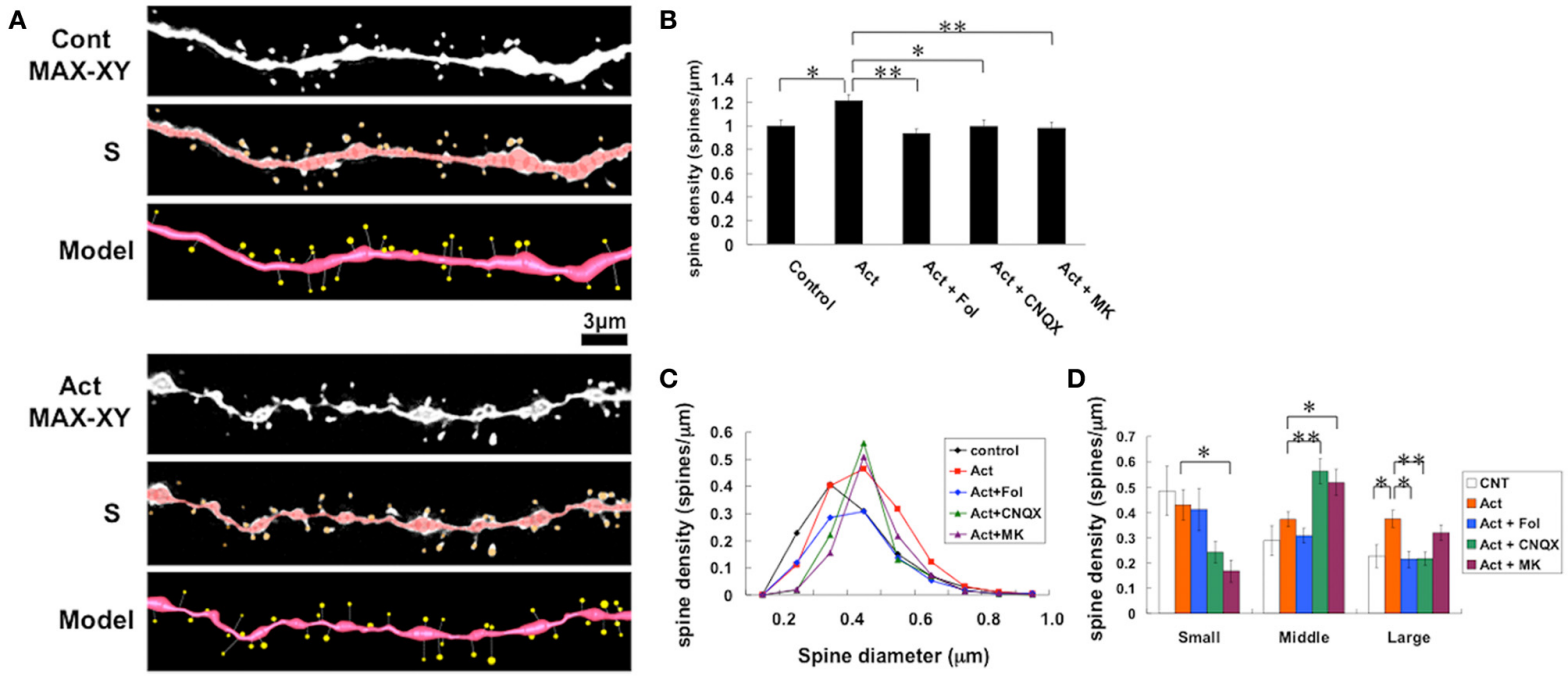
**Changes in the density and morphology of spines by activin and drugs in hippocampal slices**. Spines were analyzed along the secondary dendrites of pyramidal neurons in the stratum radiatum of CA1 neurons. **(A)** Representative images of confocal micrographs; spines along dendrite without drug-treatments (Cont) and spines along dendrite after activin treatment for 2 h (Act). Maximal intensity projection onto XY plane from z-series confocal micrographs (MAX-XY), image analyzed by Spiso-3D (S) and 3 dimensional model (Model) are shown together. Bar, 3 μm. **(B)** Effect of treatments by activin and glutamate receptor blockers on the total spine density in CA1 neurons. Vertical axis is the average number of spines per 1 μm. A 2-h treatment in ACSF without drugs (Control, total spine numbers = 552, 8 neurons), with 10 ng/mL activin A (Act), with 10 ng/mL activin and 100 ng/mL follistatin (Act + Fol), with 10 ng/mL activin and 20 μM CNQX (Act + CNQX, total spine numbers = 977, 11 neurons, *P* = 0.005), with 10 ng/mL activin and 50 μM MK-801 (Act + MK). Statistical significance is calculated against activin treated group and indicated by stars. ^*^P < 0.05, ^**^P < 0.01. **(C)** Histogram of spine head diameters after a 2 h treatment in ACSF without drugs (Control, closed black diamond), with 10 ng/mL activin (closed red square), and with 10 ng/mL activin A and 100 ng/mL follistatin (closed blue diamond), with 10 ng/mL activin and 20 μM CNQX (closed green triangle), with 10 ng/mL activin A and 50 μM MK-801 (closed purple triangle). **(D)** Density of three subtypes of spines. Abbreviations are same as in **(A)**. Vertical axis is the number of spines per 1 μm of dendrite. From left to right, small-head spines (Small), middle-head spines (Middle), and large-head spines (Large) type. ACSF without drugs (open column), Act (orange column), Act + Fol (blue column), Act + CNQX (green column), Act + MK801 (purple column) are shown. Statistical significance is calculated against activin treated group in each spine subtypes and comparisons reached significance are indicated by stars. The significance yielded *P* < 0.05. ^*^P < 0.05, ^**^P < 0.01. In **(B,D)**, results are reported as mean ± s.e.m. For each drug treatment, we investigated 3 rats, 7 slices, 14 neurons, 28 dendrites and 1400–2000 spines. For control, we used 5 rats, 8 slices, 16 neurons, 31 dendrites and approx. 1700 spines.

We investigated the effect of activin A on the modulation of the dendritic spine density and morphology in the hippocampus. We analyzed secondary branches of the apical dendrites located 100–250 μm distant from the pyramidal cell body around the middle of the stratum radiatum of CA1 region (Figure 3A). A 2 h treatment with 10 ng/mL (0.4 nM) activin A increased the total spine density to 1.21 ± 0.05 spines/μ m from 1.00 ± 0.05 spines/μm (control, no activin) (Figure 3B). However, the enhancement was not significant at 1 h after activin treatment (1.05 ± 0.06 spines/μ m). Also to determine the effective activin concentration, we applied activin at 1 and 10 ng/mL. The significant effect on the total spine density was obtained at 10 ng/mL activin. Blocking of 10 ng/mL activin by 100 ng/mL follistatin completely abrogated the enhancing effect of activin on the spine density (0.94 ± 0.04 spines/μ m).

#### Spine head diameter analysis

The morphological changes in spine head diameter induced by 2 h treatments were assessed (Figures 3C,D). We classified the spines into three categories using their head diameter, i.e., 0.2–0.4 μm as small-head spines, 0.4–0.5 μm as middle-head spines, and larger than 0.5 μm as large-head spines. In control slices (0 nM activin), the spine density was 0.48 ± 0.1 spines/μ m for small-head spines, 0.29 ± 0.06 spines/μ m for middle-head spines, and 0.23 ± 0.05 spines/μm for large-head spines. Upon treatment with activin, the density of large- and middle-head spines increased significantly from 0.23 to 0.37 spines/μ m and from 0.29 to 0.37 spines/μm, respectively, while the density of small-head spines was not significantly altered (from 0.48 to 0.43 spines/μ m) (Figure 3D). The concurrent application of activin A and follistatin decreased the density of large-head spines significantly down to 0.22 spines/μ m, while the densities of other types of spines were not significantly changed (Figure 3D).

### Activin enhances spinogenesis via Erk/MAPK, PKA, and PKC pathways

Next we investigated kinase signaling pathways involved in the activin-induced spinogenesis by using specific inhibitors.

#### Total spine density analysis (Figure 4A)

Blocking Erk/MAPK, by application of 20 μ M PD98059 (Dudley et al., 1995), abolished the activin effect on the increase of spine density, resulting in 0.86 ± 0.04 spines/μm. Application of 10 μ M H89 (Chijiwa et al., 1990), a PKA (protein kinase A) inhibitor, also prevented the effect by activin resulting in 0.99 ± 0.03 spines/μm. Treatment with 10 μ M chelerythrine (CHEL) (Herbert et al., 1990), a PKC (protein kinase C) inhibitor, also prevented the effect by activin resulting in 0.98 ± 0.05 spines/μ m. Further, 1 μ M KN-93 (Sumi et al., 1991; Niki et al., 1993), a CaMKII inhibitor, abolished the effect by activin (1.00 ± 0.05 spines/μ m). Interestingly, on the other hand, 10 μ M SB203580 (SB) (Arana-Argaez et al., 2010), a p38 MAPK inhibitor, did not prevent the activin effect on spinogenesis (1.12 ± 0.06 spines/μ m). A PI3K inhibitor, 10 μ M LY294002 (LY) (Vlahos et al., 1994), did not alter the activin effect (1.20 ± 0.04 spines/μ m). One μ M cyclosporin A (CsA) (Wiederrecht et al., 1993), an inhibitor of calcineurin (PP2B), a phosphatase, reversed the effect of activin (1.04 ± 0.03 spines/μ m).

**Figure 4:**
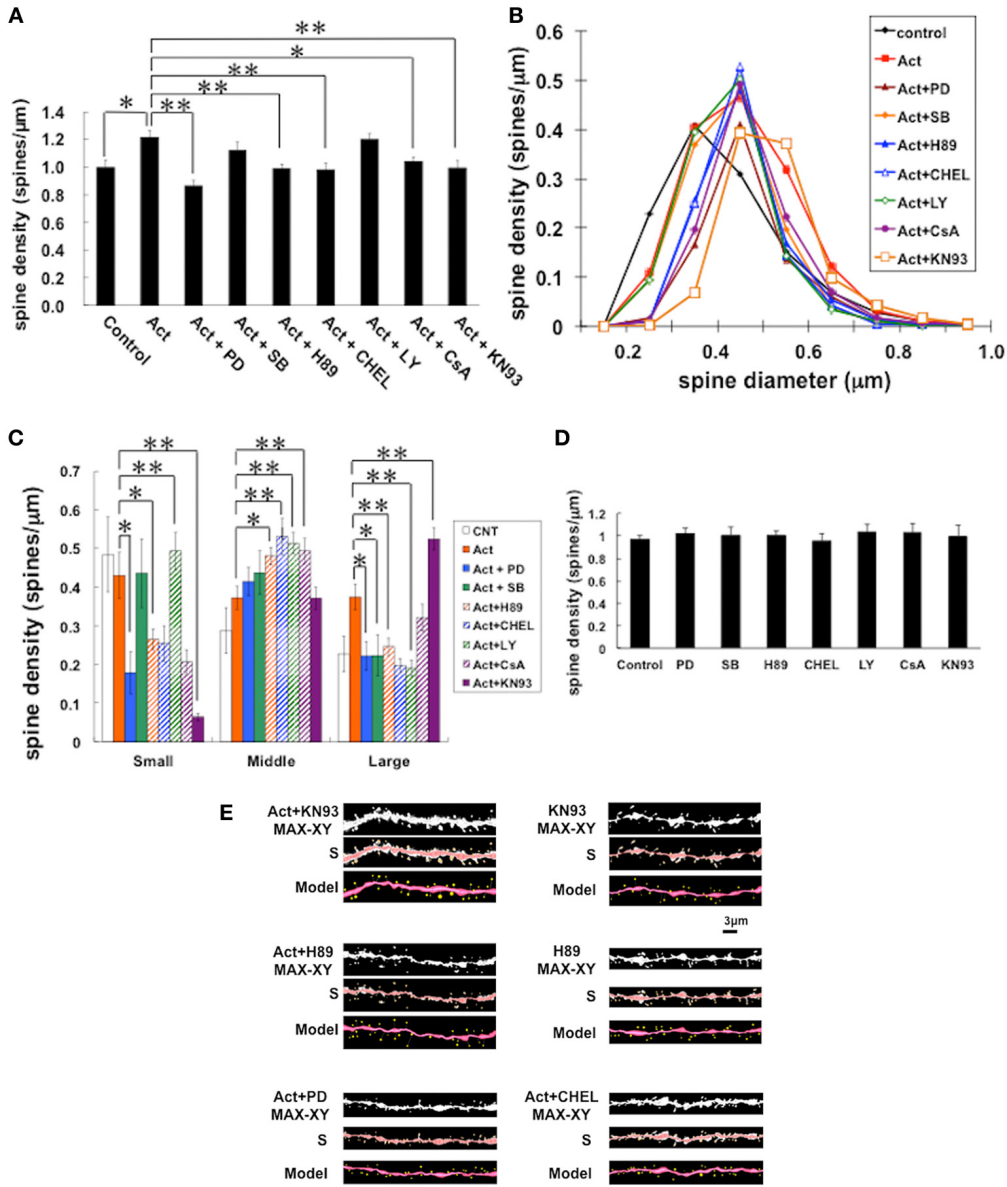
**Effects by inhibition of kinases on changes of the density and morphology of spines in the presence of activin A**. Spines were analyzed along the secondary dendrites of CA1 pyramidal neurons. **(A)** Total spine density. Effect of kinase inhibitors in the presence of activin in CA1 neurons. Vertical axis is the average number of spines per 1 μm. A 2-h treatment in ACSF without drugs (Control), with 10 ng/mL activin (Act), with 10 ng/mL activin and 20 μM PD98059 (Erk MAPK inhibitor) (Act + PD), with 10 ng/mL activin and 10 μM SB203580 (p38 MAPK inhibitor) (Act + SB), with 10 ng/mL activin and 10 μM H-89 (PKA inhibitor) (Act + H89), with 10 ng/mL activin and 10 μM chelerythrine (PKC inhibitor) (Act + CHEL), with 10 ng/mL activin A and 10 μM LY294002 (PI3K inhibitor) (Act + LY), with 10 ng/mL activin and 1 μM cyclosporin A (calcineurin inhibitor) (Act + CsA), and with 10 ng/mL activin and 1 μM KN-93 (CaMKII inhibitor) (Act + KN93). Statistical significance is calculated against activin treated group and indicated by stars. ^*^P < 0.05, ^**^P < 0.01. **(B)** Histogram of spine head diameters. Abbreviations are the same as in **(A)**. Vertical axis is the number of spines per 1 μm of dendrite. After a 2-h treatment in ACSF without drugs (Control, closed black diamond), Act (closed red square), Act + PD (closed brown triangle), Act + SB (closed orange diamond), with Act + H89 (closed blue triangle), with Act + CHEL (open blue triangle), with Act + LY (open green diamond), and Act + CsA (closed purple circle), and Act + KN93 (open orange square). **(C)** Density of three subtypes of spines. Abbreviations are the same as in **(A)**. Vertical axis is the average number of spines per 1 μm of dendrite. From left to right, small-head spines (Small), middle-head spines (Middle), and large-head spines (Large). In each group, control (open column), Act (closed orange column), Act + PD (closed blue column), Act + SB (closed green column), Act + H89 (hatched orange column), Act + CHEL (hatched blue column), Act + LY (hatched green column), and Act + CsA (hatched purple column), and Act + KN93 (closed purple column). Statistical significance is calculated against activin treated group in each spine subtypes and comparisons reached significance are indicated by stars. The significance yielded *P* < 0.05. ^*^P < 0.05, ^**^P < 0.01. **(D)** No effect of kinase inhibitors alone on the total spine density in CA1 neurons. Abbreviations are same as in **(A)**. **(E)** Representative spine images of confocal micrographs used for **(A–C)**: activin plus KN-93 treatment (Act+KN93) and only KN-93 treatment (KN93); activin plus H-89 treatment (Act+H89) and only H-89 treatment (H89); activin plus PD98059 treatment (Act+PD); activin plus chelerythrine treatment (Act+CHEL). Maximal intensity projection onto XY plane from z-series (MAX-XY), image analyzed by Spiso-3D (S) and 3 dimensional model (Model) are shown together. Bar, 3 μm. In **(A,C,D)**, results are reported as mean ± s.e.m. For each drug treatment, we investigated 3 rats, 7 slices, 14 neurons, 28 dendrites and 1400–2000 spines. For control, we used 5 rats, 8 slices, 16 neurons, 31 dendrites and approx. 1700 spines.

It should be noted that these kinase inhibitors alone did not significantly affect the total spine density within experimental error, indicating that the observed inhibitory effects are not due to simple blockers' effects (Figure 4D).

#### Spine head diameter analysis (Figures 4B,C,E)

Since the total spine density is not sensitive enough to describe complex effects of kinases, the changes in spine head diameter distribution should be analyzed. In inhibitory effect by MAPK inhibitor, middle-head spines remained unchanged, while smaller and larger population of spines showed considerable decrease (Figures 4B,C,E). Even though the total spine density was not altered by the treatment of LY or SB in the presence of activin, the density of small-, middle- or large-head spines changed.

In order to statistically analyze these complicated morphological alterations, we classified the spine head into three categories depending on their head diameters (Figures 4C,E). Categorizing spines by two classes (for example, thin or mushroom) was not sufficient to describe the complex changes of spine heads. Blocking Erk/MAPK (PD) abolished the effect of activin on the spine densities, decreasing the density of large-head spines from 0.37 to 0.22 spines/μm and small-head spines from 0.43 to 0.18 spines/μm, while significant change was not observed in middle-head spines (from 0.37 to 0.41 spines/μm). Inhibiting PKA (H89) also decreased the density of large-head spines from 0.37 to 0.25 spines/μm and small-head spines from 0.43 to 0.27 spines/μm, with an increase in middle-head spines (from 0.37 to 0.48 spines/μ m). Inhibiting PKC (CHEL) had a similar effect to that of PKA, decreasing the density of large-head spines from 0.37 to 0.20 spines/μm and small-head spines from 0.43 to 0.25 spines/μ m, with an increase in middle-head spines (from 0.37 to 0.53 spines/μ m).

### Effect of other kinases on activin-induced spinogenesis

Although inhibition of p38 MAPK and PI3K did not significantly change the total spine density, these inhibitors altered subpopulations of spines. Inhibition of p38 MAPK (SB) decreased the density of large-head spines, but increased middle-head spines without changing small-head spines. PI3K inhibitor (LY) decreased the density of large-head spines, however, increased middle-head spines and small-head spines. On the other hand, although total density of spines was reversed by blocking of CaMKII (KN93), KN93 increased large-head spines from 0.37 to 0.53 spines/μm, but it decreased small-head spines, with no significant changes in middle-head spines (Figures 4B,C). Inhibition of calcineurin by CsA decreased small-head spines, increased middle-head spines, without a change in large-head spines.

### Blocking of glutamate receptors abolished activin-induced spinogenesis

We investigated the contribution of ionotropic glutamate receptors to activin effects. The level of Ca^2+^ or Na^+^, which is maintained via ionotropic glutamate receptors, may be essential for activin-induced spinogenesis. We examined spinogenesis in the presence of inhibitors of these receptors. CNQX, an inhibitor of AMPA receptor, and MK-801, an NMDA receptor blocker, suppressed the activin effects on the total spine density (Figure 3B). Note that these inhibitors alone did not alter the density of spines.

The morphological changes in spine head size in the presence of glutamate receptor inhibitors were analyzed (Figures 3C,D). CNQX decreased the density of large-head spines from (from 0.37 to 0.21 spines/μm) and small-head spines (from 0.43 to 0.24 spines/μ m), but increased middle-head spines (from 0.37 to 0.56 spines/μ m). MK-801 only slightly decreased large-head spines, but considerably decreased small-head spines, and increased middle-head spines.

### Activin-induced spinogenesis is dependent on synthesis of protein or mRNA

**Figure 5:**
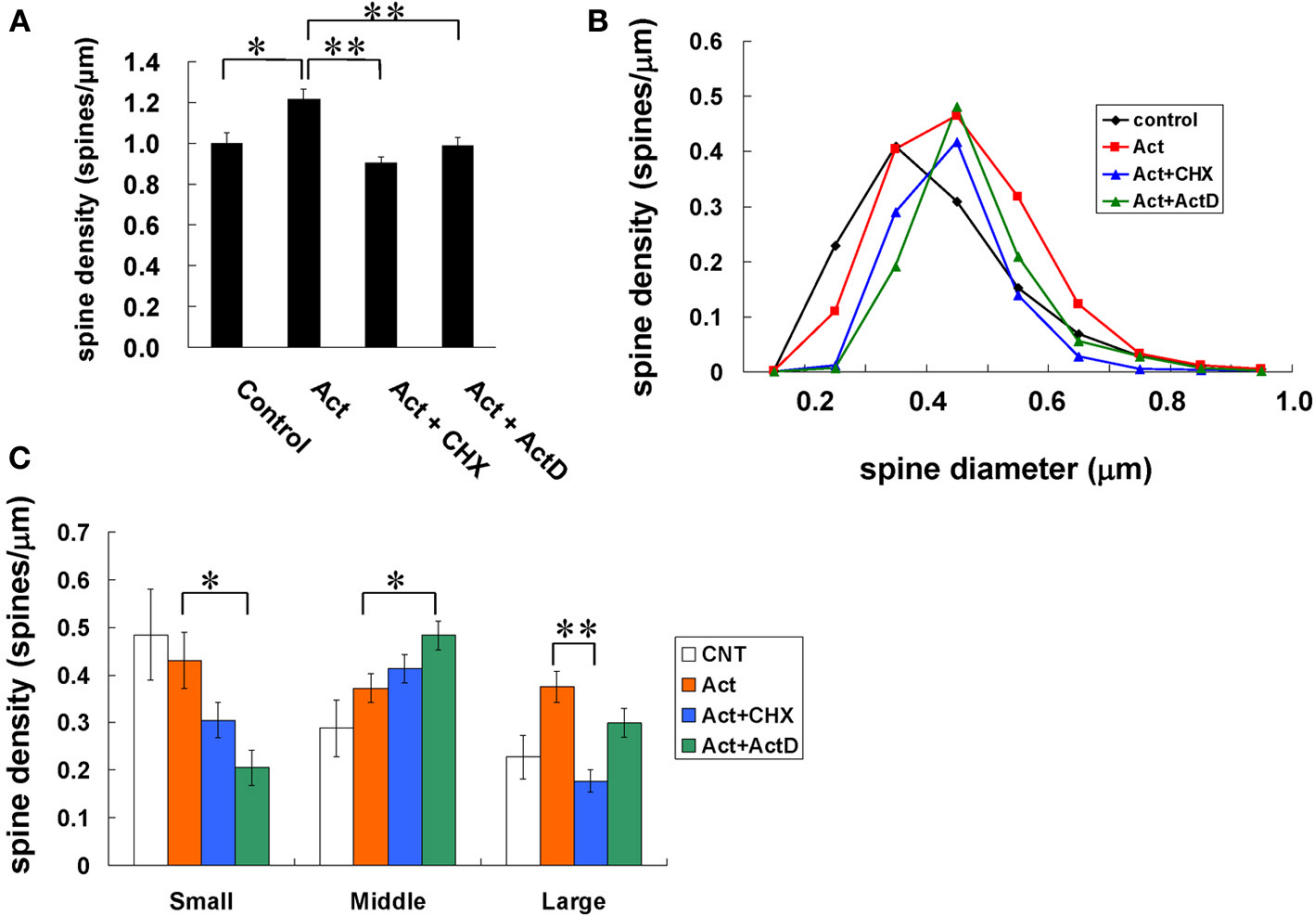
**Effects of protein and mRNA synthesis on changes in the density and morphology of spines by activin**. Spines were analyzed along the secondary dendrites of CA1 pyramidal neurons as in Figure 3. **(A)** Total spine density. Effect of inhibitors for protein or mRNA synthesis in the presence of activin on CA1 neurons. Vertical axis is the average number of spines per 1 μm. A 2-h treatment in ACSF without drugs (Control), with 10 ng/mL activin (Act), with 10 ng/mL activin and 20 μM cycloheximide (Act + CHX), and with 10 ng/mL activin and 4 μM actinomycin D (Act + actD). Statistical significance is calculated against activin treated group. ^*^P < 0.05, ^**^P < 0.01. **(B)** Histogram of spine head diameters. Abbreviations are same as in **(A)**. Vertical axis is the number of spines per 1 μm of dendrite. After a 2-h treatment in ACSF without drugs (Control, closed black diamond), Act (closed orange square), Act + CHX (closed blue triangle), and Act + ActD (closed green triangle). **(C)** Density of three subtypes of spines. Abbreviations are same as in **(A)**. Vertical axis is the average number of spines per 1 μm of dendrite. From left to right, small-head spines (Small), middle-head spines (Middle), and large-head spines (Large). ACSF without drugs (open column), Act (orange column), Act + CHX (blue column), Act + actD (green column). Statistical significance is calculated against activin treated group in each spine subtypes and comparisons reached significance are indicated by stars. The significance yielded *P* < 0.05. ^*^P < 0.05, ^**^P < 0.01. In **(A,C)** results are reported as mean ± s.e.m. For each drug treatment, we investigated 3 rats, 6 slices, 12 neurons, 24 dendrites and 1100–1800 spines. For control, we used 5 rats, 8 slices, 16 neurons, 31 dendrites and approx. 1700 spines.

To investigate whether activin-induced spinogenesis includes new protein recruitment, we examined synthesis of protein or mRNA (Figure 5). Cycloheximide (CHX), a protein synthesis inhibitor, at 20 μM abolished the activin-induced increase in the density of spines, by decreasing the total density to 0.90 spines/μm. Actinomycin D (actD), an mRNA synthesis inhibitor, at 4 μ M also suppressed the effect of activin. These inhibitors alone did not alter the density of spines. Upon CHX treatment, the density of large-head spines and small-head spines were decreased, without a significant change in middle-head spines (Figure 5). On the other hand, actD slightly decreased large-head spines, and considerably decreased small-head spines, but increasing middle-head spines.

### Activin induces moderate LTP upon weak-TBS (15 pulses)

**Figure 6:**
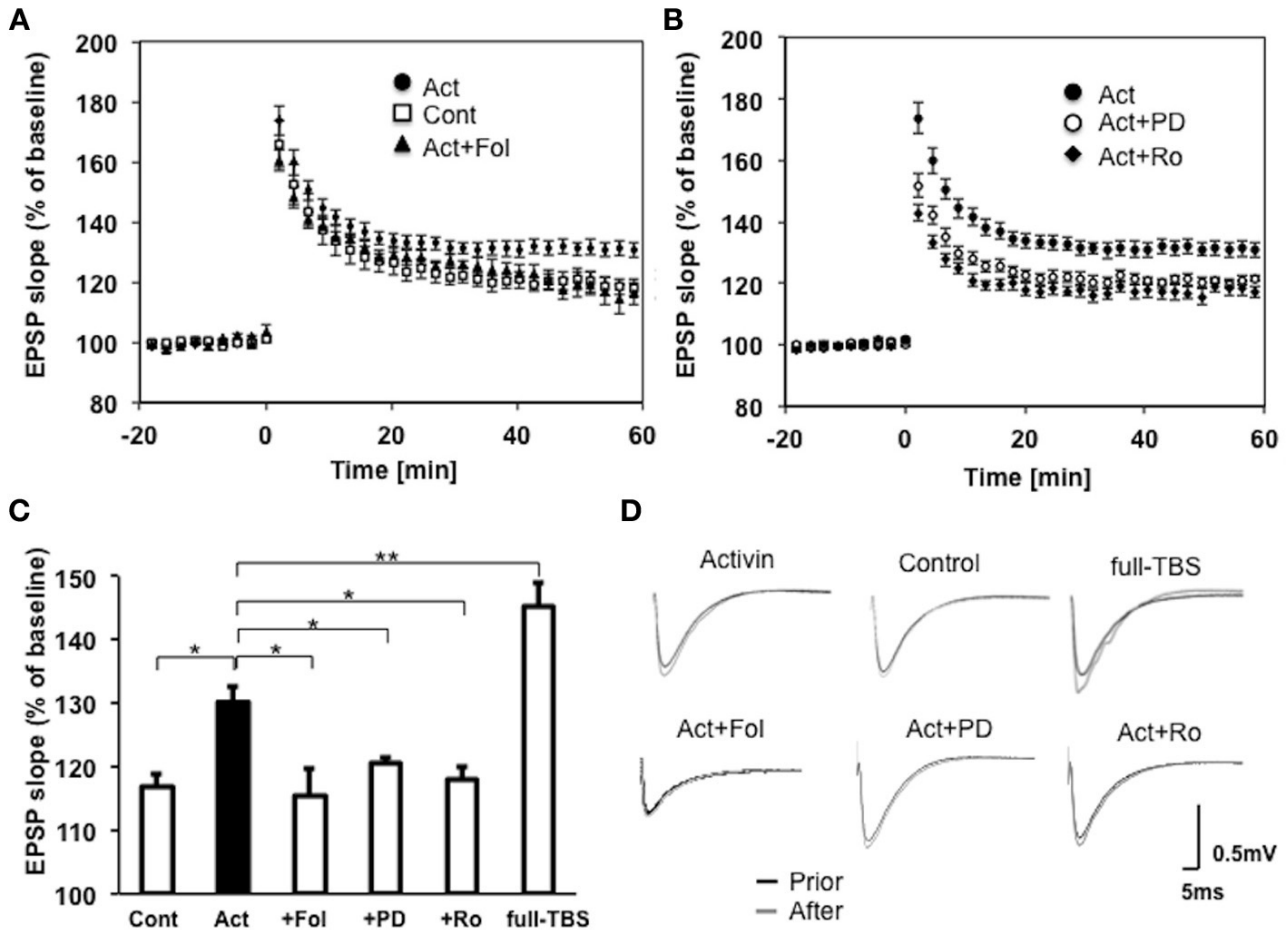
**(A)** Induction of moderate LTP by weak-TBS stimulation after short incubation (~2 h) with activin in the CA1 of hippocampal slices. Slices with 0 ng/ml activin (control, open square, *n* = 10 slices, 10 rats), with 10 ng/ml activin (closed circle, *n* = 10 slices, 10 rats), with 10 ng/ml activin plus 100 ng/ml follistatin (closed triangle, *n* = 7 slices, 7 rats), with respectively. The number of independent experiments is indicated as *n*. Vertical axis indicates EPSP slope. Here, 100% refers to the EPSP slope value of the average of *t* = −10 to −1 min prior to weak-TBS stimulation. LTP was induced at time *t* = 0. Illustrated data points and error bars represent the mean ± s.e.m. from n of independent slices. **(B)** Co-incubation of activin with MAPK inhibitor PD98059 (20 μ M) prevented the induction of LTP (open circle, *n* = 7 slices, 7 rats). Co-incubation of activin with NR2B inhibitor Ro25-6981 (1 μ M) prevented the induction of LTP (open square, *n* = 7 slices, 7 rats). Activin-treated slices (closed circle, *n* = 10 slices, 10 rats). Maximal LTP by full-TBS is also shown (closed circle, *n* = 7, 7 rats). **(C)** Comparison of modulation effects by activin upon weak-TBS as shown in **(A)** and **(B)**. From left to right; slices without drugs (Cont), with 10 ng/ml activin (Act), with activin plus follistatin (+Fol), activin plus PD98059 (+PD), activin plus Ro25-6981 (+Ro) and full-TBS (full-TBS). The significance yielded *p* < 0.05. ^*^P < 0.05, ^**^P < 0.01. **(D)** Representative raw traces of EPSP, showing sample recordings prior to (black line) or after (gray line) weak-TBS stimulation. Control (0 ng/ml activin), Act (10 ng/ml activin), Act+ PD (activin plus PD98059), Act + Ro (activin plus Ro25-6981). EPSP trace for full-TBS is also shown.

To investigate the effect of activin incubation on the synaptic transmission, we measured LTP upon weak-TBS in CA1 region of the adult hippocampal slices. Acute slices were incubated for 2 h with 10 ng/ml (0.4 nM) activin before weak TBS (15 pulses). The activin treatment established moderate LTP-induction upon weak-TBS, by increasing the magnitude of EPSP from 119 ± 3 % (small LTP, *n* = 10 slices) in control slices (with no activin) to 130 ± 2 % (moderate LTP, *n* = 10 slices) in activin-treated slices (Figures 6A,C). Co-incubation of follistatin (100 ng/ml), an endogenous inhibitor of activin, completely blocked the effect of activin, resulting in LTP magnitude of 116 ± 4 % (*n* = 7 slices) (Figures 6A,C). Note that full-TBS (with total 50 pulses) elevated the EPSP to 145 ± 4 (full LTP, *n* = 9 slices), implying that the activin-induced moderate LTP (130%) is smaller than full LTP (145%) (Figure 6C).

#### Erk/MAP kinase inhibitor prevents activin-induced LTP

Co-incubation of PD98059 (20 μ M), an inhibitor of Erk/MAPK, with activin considerably suppressed the effect of activin, resulting in LTP magnitude of 121 ± 1 % (*n* = 7 slices) (Figures 6B,C). Note that incubation with only PD98059 did not change the small LTP upon weak TBS (117 ± 4 %, *n* = 5 slices).

#### NR2B inhibitor prevents activin-induced LTP

Co-incubation of Ro25-6981 (1 μ M) (Ooishi et al., 2012b), an inhibitor of NR2B subunit of NMDA receptors, with activin considerably suppressed the effect of activin A, resulting in LTP magnitude of 118 ± 2 % (*n* = 7 slices) (Figures 6B,C). Note that incubation with only Ro25-6981 did not change the small LTP upon weak TBS (122 ± 5 %, *n* = 5 slices).

### Determination of endogenous level of activin

Endogenous level of activin was quantified by ELISA. Brains were taken out after deep anesthesia and each brain region was dissected out. Tissues were homogenized in the buffer [0.32 M sucrose, 5 mM Tris-HCl, pH 8.0, protease inhibitor cocktail (Roche)], and homogenates were centrifuged at 20,000 × g at 4°C. Activin level was measured by ELISA kit (Quantikine Activin A assay, R&D systems) according to manufacturer's instruction. Approximately same concentration of activin (5 ng/mL) in the hippocampus is obtained with previous studies using wild type mice (Ageta et al., 2008).

## Discussion

**Figure 7:**
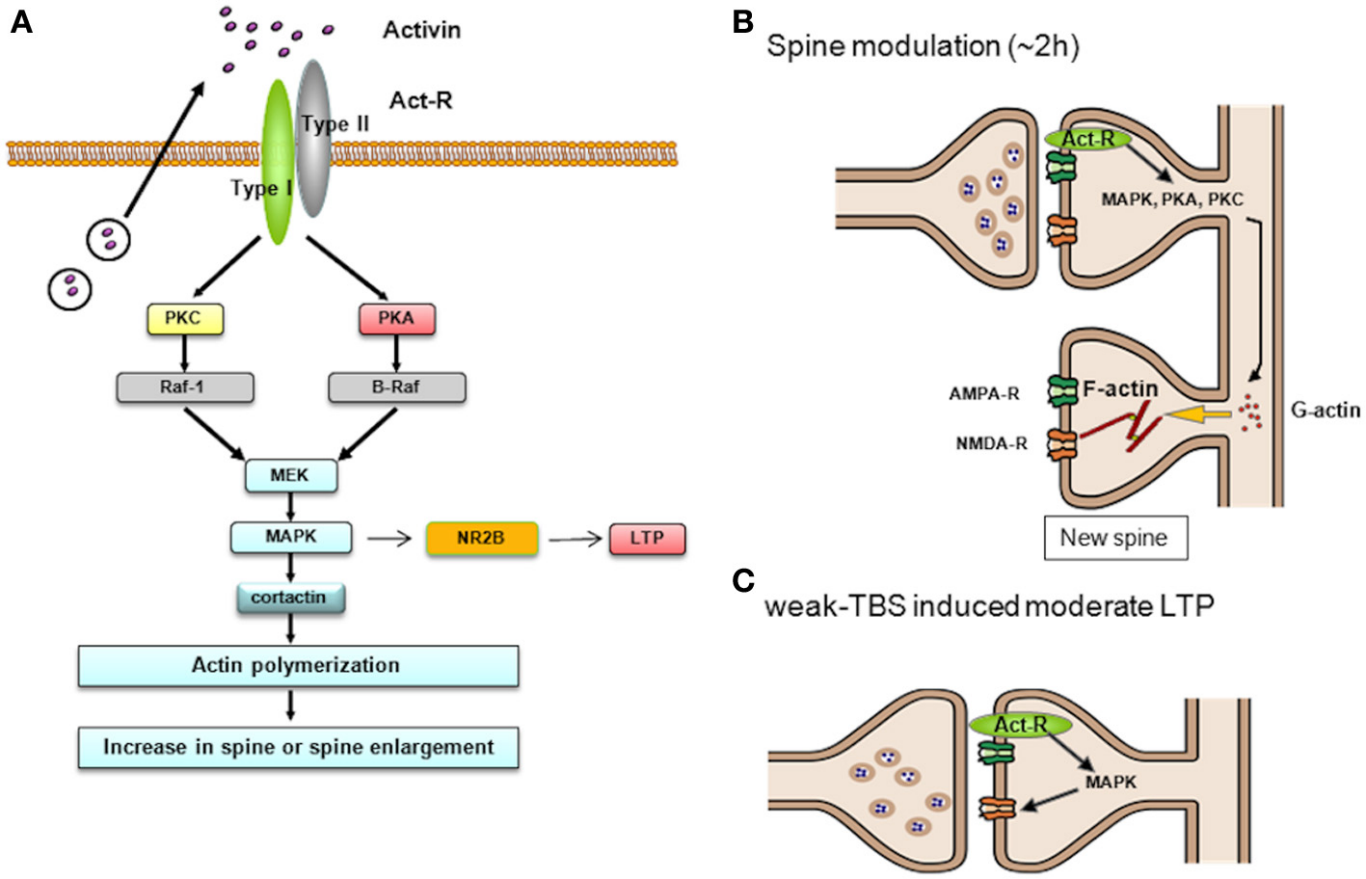
**Schematic illustration of activin-induced spinogenesis via multiple kinase pathways. (A)** Upon activation by activin receptor complex (ActR), early LTP is suppressed by anti-inflammatory action of activin. **(B)** After 2 h of ActR activation, PKA, PKC and MAPK may promote actin polymerization process, leading to the formation of new spines. **(C)** After 2 h of ActR activation, MAPK may phosphorylate NR2B of NMDA receptors, thereafter moderate LTP is induced upon weak-TBS.

We examined the role of activin in an acute effect on adult rat hippocampus under physiological conditions. To date much effort has been devoted to investigate the role of activin as neuroendocrinological factor in hypothalamic-pituitary-gonadal (HPG) axis (Ling et al., 1986; Vale et al., 1986; Gregory and Kaiser, 2004) or neuroprotective factor in excitotoxic injury (Tretter et al., 1996, 2000). We attempt to find physiological roles in addition to pathological roles of activin in the hippocampus (see model illustration of Figure 7). Activin drives a variety of kinases (including Erk/MAPK, PKA and PKC), resulting in modulation of spine density and spine head shape. Importantly, some kinases including p38 MAPK and PI3K, did not participate in these actions, indicating that the observed kinase effects are not non-specific ones.

We used exogenous activin-treatments in the current work in order to simulate the physiological elevation in activin level in the hippocampus. Since the acute change in the activin level in slices is difficult with other methods, these exogenous application is also used in other studies for investigations of the role for endogenous activin (Ikegaya et al., 1997; Tretter et al., 2000; Shoji-Kasai et al., 2007; Kurisaki et al., 2008; Ageta et al., 2010). Applied concentration of activin in the current study (10 ng/mL = 0.4 nM) is comparable with endogenous level of activin found in the hippocampus (5 ng/mL = 0.2 nM) (Ageta et al., 2008).

### Activin receptors localize within synapse

Activin has synaptic actions. In primary cultured hippocampal neurons, activin induces rapid activation (~1 h) of NMDA receptors (e.g., elevation of Ca^2+^ influx) via Fyn-kinase dependent phosphorylation of NR2A or NR2B (Kurisaki et al., 2008). This activin-induced activation of NMDA receptors is not blocked by SB431542, an inhibitor of Smad phosphorylation, indicating that this pathway is Smad-independent. Transgenic mice expressing dominant-negative activin receptor IB exhibit reduced NMDA current and impaired LTP at the Schaffer-CA1 synapse (Muller et al., 2006). The synaptic action of activin is further supported by the synaptic distribution of activin type IB receptor, which is associated with PSD regions (containing both NMDA receptors and Erk/MAPK) (see Figure 5). Activin type I receptor was expressed in hippocampal CA1, and CA3 pyramidal neurons, in addition to the receptor distribution in DG (Funaba et al., 1997). The synaptic activin receptor probably plays an essential role in rapid signaling, since the activin-induced modulation of spine morphology and LTP appeared within 2 h.

### Activin enhances spinogenesis via Erk/MAPK, PKA, and PKC and their downstream under physiological conditions

Upon inhibition of Erk/MAPK, PKA, or PKC, the activin-induced enhancement of spinogenesis was significantly prevented. Non-specific effects by only kinase inhibitors were not observed (Figure 4D). Detailed analysis of spine head diameter revealed that inhibition of Erk/MAPK, PKA, or PKC decreased density of large-head spines. Analysis of individual spine head diameters using the criteria of small, middle, and large classes (using three classes), was particularly useful to distinguish different effects of many kinases on the activin-induced spinogenesis as compared with previous classification of mushroom or thin (only two classes).

Not only Erk/MAPK but also PKA and PKC might also contribute in reorganization of spines. Importantly in CA1, MAPK cascade is known to couple with PKA and PKC via PKC → Raf1 → MAPK and/or PKA → B-Raf → MAPK in synaptic modulation including LTP (Roberson et al., 1999). Taking this knowledge into account, MAPK may be a key kinase responsible for modulation of spines.

What is the target of Erk/MAPK in spine reorganization? Erk/MAPK is known to phosphorylate cortactin, a structural protein associated to actin (Campbell et al., 1999; Macqueen et al., 2003). Cortactin interacts with both F-actin and actin-related protein (Arp) 2/3 complex as well as scaffold protein Shank in the PSD at the SH3 domain (Weed et al., 1998; Campbell et al., 1999), resulting in promotion of actin fiber remodeling within spines. As a good example, upon BDNF stimulation, MAPK phosphorylates cortactin through interacting C-terminal of SH3 domain, resulting in a reorganization of spine morphology (Iki et al., 2005).

It is thus probable that activin exerts its effect on spines via cortactin-actin pathway. Cortactin has multiple phosphorylation sites, including Ser^405^ and Ser^418^, which are activated by MAPK (Campbell et al., 1999). Phosphorylation of cortactin may promote assembly of actin cytoskeletal matrices, resulting in spine formation or modulation of spine morphology (Hering and Sheng, 2003). These sites, including Ser^113^,are putative phosphorylation sites also for other serine/threonine kinase (PKA or PKC) that are activated by activin.

LIMK might also involve in the regulation of spinogenesis through Rho-ROCK pathway. It has been known that TGFbeta family receptors regulate the phospharylation of LIMK (Bernard, 2007).

Accumulating evidence indicates that Erk/MAPK is a rapid, Smad-independent signaling pathway of TGF family receptors, including activin (Ten Dijke et al., 2000; Derynck and Zhang, 2003; Lee et al., 2007). Via interaction with Ras, TGF family receptors induce phosphorylation of Erk/MAPK within 2 h (Lee et al., 2007). On the other hand, Smad pathway via gene transcription (Derynck and Zhang, 2003) typically needs longer hours. For example, phosphorylation of Smad2 requires 4 h after induction of activin expression by acute electroconvulsive shock treatment (Dow et al., 2005).

Figure 4A shows the inhibition effects of kinases, including CaMKII, in the basal low Ca conditions (around 0.1–0.2 μ M). In this low Ca level, CaMKII is not highly activated (Pi and Lisman, 2008), and CaMKII action should be very different from that in the high Ca level (~10 μ M). In earlier studies (Yuste and Bonhoeffer, 2001; Lee et al., 2009; Hamilton et al., 2012), activity-dependent spine growth effects (spine size enlargement) by CaMKII have been observed in which high level Ca influx occurs (through NMDA receptors) upon stimulation by glutamate or high frequency electric stimulation. Due to the low Ca condition, Figure 4 results suggest new and interesting aspects. For example, CaMKII may maintain the shorter F-actin filaments, therefore by blockade of CaMKII, F-actin filaments might be elongated, resulting in significant conversion from small head to middle head and from middle head to large head spines. From similar considerations, the PKA/PKC/Erk MAPK may maintain longer F-actin filaments, therefore by blockade of PKA/PKC/Erk MAPK under activin treatments, large spines (with long F-actin filaments) may be converted to middle spines, and small spines were lead to disappear. KN93 effects may not be solely attributed to CaMKII, since KN93 may also inhibit other CaMKs.

### Contribution of NMDA receptors and RNA/protein synthesis

Blockade of NMDA receptors by MK-801 inhibited the activin-induced increase of the total density of spines (Figure 3). MK-801 probably further decreased the Ca level below the basal Ca level by suppression of Ca exchange through spontaneous opening/closing of NMDA receptors. By the presence of MK-801, middle-head spines were increased and small-head spines were considerably decreased. Decrease of Ca level to below the basal level may shrink small spines, leading to the disappearance of small spines, and may convert large-head spines to middle head spines, by suppressing PKA/PKC/Erk MAPK functions which might enlarge spines (Figure 4). Since inhibition of new protein synthesis by CHX as well as inhibition of gene transcription by actD abolished activin-induced spinogenesis (Figure 5), rapid transcription/translation might also contribute to spinogenesis.

### Activin induces moderate LTP upon weak-TBS

The current study demonstrates that activin-treatments induced moderate LTP upon weak-TBS (Figure 6). Induction of moderate LTP upon weak-TBS stimuli by the presence of neurotrophic factors such as BDNF or estradiol is observed (Kramar et al., 2004, 2009). On the other hand these neurotrophic factors fail to further enhance strong tetanus-induced LTP (Ooishi et al., 2012b), probably because strong tetanus stimulation (typically 100 Hz, 1 s) elevates EPSP to a saturated level which cannot be further enhanced by neurotrophic factors. Activin-treatments also could not further enhance the full LTP induced by full TBS in our study.

The rapid action of activin is driven via Erk/MAPK, because PD98059 completely suppressed the activin-induced moderate LTP. Since Erk/MAPK is localized in spines and the PSD fractions (Mukai et al., 2007), the activation of Erk/MAPK may phosphorylate tyrosine of NR2B as shown in the case of estradiol-induced MAPK signaling (Dominguez et al., 2007) or leptin-induced MAPK signaling (Irving et al., 2006). The involvement of NR2B is supported by the observation that NR2B inhibitor Ro25-6981 suppressed the activin-induced LTP. The rapid phosphorylation of NR2B by activin is found in hippocampal neurons (Kurisaki et al., 2008). We therefore hypothesize that the signaling pathway is as follows: activin → type II receptor → Erk/MAP kinase → activation of NMDA receptor by phosphorylation of NR2B → increase of Ca^2+^ influx though NMDA receptors during weak-TBS → enhanced phosphorylation of AMPA receptors → enhancement of the magnitude of LTP (Figure 7).

### Physiological and pathological functions of activin in earlier studies

We found a significant endogenous expression of activin molecules in hippocampal glutamatergic neurons in CA1, CA3, and DG region, along with the expression of activin mRNA. A much less expression of activin A was observed in glial cells.

To date, activin expression and neuroprotective effects upon excitotoxic injury have been extensively investigated (Tretter et al., 1996, 2000). Electroconvulsive shock shows upregulation of activin mRNA in the hippocampus (Inokuchi et al., 1996; Dow et al., 2005). It is suggested that local upregulation of endogenous activin A may be of crucial importance for neuroprotection in hippocampal neurons (Iwahori et al., 1997; Tretter et al., 2000).

In addition to above neuroprotective roles of activin, recent evidence suggests physiological roles of activin in the brain. Activin modulates the long term memory and late-LTP *in vivo* (Ageta et al., 2010). Mice overexpressing follistatin have a severe deficit in adult hippocampal neurogenesis that affects maintenance of fear memory (Kitamura et al., 2009). Current study adds further importance of physiological function of activin about synaptic plasticity. Significant expression of activin and its receptors in healthy neurons of CA1, CA3 and DG supports physiological role of activin. Its role may be regulation of neuronal plasticity, including enhancement of spinogenesis or LTP under moderate stimulation (weak-TBS). Further efforts are needed to unravel a possible underlying mechanism of activin to enhance memory processes in the hippocampus in physiological conditions.

### Similarity to estradiol-induced spinogenesis

Comparison between activin and 17β-estradiol about spinogenesis is interesting, since these two sex-hormones are synthesized in the hippocampus. Estradiol induced rapid increase in spines by approx. 1.4-fold in the adult male hippocampus and this increase was inhibited by Erk MAPK inhibitor (Mukai et al., 2007). Both activin- and estradiol-induced spinogenesis was suppressed by NMDA receptor blocking.

### Conflict of interest statement

The authors declare that the research was conducted in the absence of any commercial or financial relationships that could be construed as a potential conflict of interest.

## Supplementary material

Figure S1 **(A)** Western blot of activin receptor and activin. ActR, activin receptor. **(B)** Act, activin; Marker, protein marker. Anti-activin antiserum recognized the main band of 13 kDa activin protein.

## References

[B1] Ageta H. Ikegami S. Miura M. Masuda M. Migishima R. Hino T. (2010). Activin plays a key role in the maintenance of long-term memory and late-LTP. Learn. Mem. 17, 176–185 10.1101/lm.16659010 20332189

[B2] Ageta H. Murayama A. Migishima R. Kida S. Tsuchida K. Yokoyama M. (2008). Activin in the brain modulates anxiety-related behavior and adult neurogenesis. PLoS ONE 3:e1869 10.1371/journal.pone.0001869 18382659 PMC2270335

[B3] Arana-Argaez V. E. Delgado-Rizo V. Pizano-Martinez O. E. Martinez-Garcia E. A. Martin-Marquez B. T. Munoz-Gomez A. (2010). Inhibitors of MAPK pathway ERK1/2 or p38 prevent the IL-1{beta}-induced up-regulation of SRP72 autoantigen in Jurkat cells. J. Biol. Chem. 285, 32824–32833 10.1074/jbc.M110.121087 20729213 PMC2963399

[B4] Asashima M. Nakano H. Uchiyama H. Davids M. Plessow S. Loppnow-Blinde B. (1990). The vegetalizing factor belongs to a family of mesoderm-inducing proteins related to erythroid differentiation factor. Naturwissenschaften 77, 389–391 10.1007/BF01135742 2274069

[B4a] Bernard O. (2007). Lim kinases, regulators of actin dynamics. Int. J. Biochem. Cell Biol. 39, 1071–1076 10.1016/j.biocel.2006.11.011 17188549

[B5] Campbell D. H. Sutherland R. L. Daly R. J. (1999). Signaling pathways and structural domains required for phosphorylation of EMS1/cortactin. Cancer Res. 59, 5376–5385 10537323

[B6] Chijiwa T. Mishima A. Hagiwara M. Sano M. Hayashi K. Inoue T. (1990). Inhibition of forskolin-induced neurite outgrowth and protein phosphorylation by a newly synthesized selective inhibitor of cyclic AMP-dependent protein kinase, N-[2-(p-bromocinnamylamino)ethyl]-5-isoquinolinesulfonamide (H-89), of PC12D pheochromocytoma cells. J. Biol. Chem. 265, 5267–5272 2156866

[B7] Derynck R. Zhang Y. E. (2003). Smad-dependent and Smad-independent pathways in TGF-beta family signalling. Nature 425, 577–584 10.1038/nature02006 14534577

[B8] Dominguez R. Liu R. Baudry M. (2007). 17-Beta-estradiol-mediated activation of extracellular-signal regulated kinase, phosphatidylinositol 3-kinase/protein kinase B-Akt and N-methyl-D-aspartate receptor phosphorylation in cortical synaptoneurosomes. J. Neurochem. 101, 232–240 10.1111/j.1471-4159.2006.04360.x 17250656 PMC3182115

[B9] Dow A. L. Russell D. S. Duman R. S. (2005). Regulation of activin mRNA and Smad2 phosphorylation by antidepressant treatment in the rat brain: effects in behavioral models. J. Neurosci. 25, 4908–4916 10.1523/JNEUROSCI.5155-04.2005 15901772 PMC6724846

[B10] Duan H. Wearne S. L. Morrison J. H. Hof P. R. (2002). Quantitative analysis of the dendritic morphology of corticocortical projection neurons in the macaque monkey association cortex. Neuroscience 114, 349–359 10.1016/S0306-4522(02)00305-6 12204204

[B11] Dudley D. T. Pang L. Decker S. J. Bridges A. J. Saltiel A. R. (1995). A synthetic inhibitor of the mitogen-activated protein kinase cascade. Proc. Natl. Acad. Sci. U.S.A. 92, 7686–7689 10.1073/pnas.92.17.7686 7644477 PMC41210

[B12] Fukui A. Komazaki S. Miyoshi O. Asashima M. (2003). Immunocytochemical study of activin type IB receptor (XALK4) in Xenopus oocytes. Dev. Growth Differ. 45, 113–119 10.1034/j.1600-0854.2004.00680.x 12752499

[B13] Funaba M. Murata T. Fujimura H. Murata E. Abe M. Torii K. (1997). Immunolocalization of type I or type II activin receptors in the rat brain. J. Neuroendocrinol. 9, 105–111 10.1046/j.1365-2826.1997.00558.x 9041363

[B14] Gregory S. J. Kaiser U. B. (2004). Regulation of gonadotropins by inhibin and activin. Semin. Reprod. Med. 22, 253–267 10.1055/s-2004-831901 15319828

[B15] Hamilton A. M. Oh W. C. Vega-Ramirez H. Stein I. S. Hell J. W. Patrick G. N. (2012). Activity-dependent growth of new dendritic spines is regulated by the proteasome. Neuron 74, 1023–1030 10.1016/j.neuron.2012.04.031 22726833 PMC3500563

[B16] Herbert J. M. Augereau J. M. Gleye J. Maffrand J. P. (1990). Chelerythrine is a potent and specific inhibitor of protein kinase C. Biochem. Biophys. Res. Commun. 172, 993–999 10.1016/0006-291X(90)91544-3 2244923

[B17] Hering H. Sheng M. (2003). Activity-dependent redistribution and essential role of cortactin in dendritic spine morphogenesis. J. Neurosci. 23, 11759–11769 14684878 10.1523/JNEUROSCI.23-37-11759.2003 PMC6740953

[B18] Hojo Y. Hattori T. A. Enami T. Furukawa A. Suzuki K. Ishii H. T. (2004). Adult male rat hippocampus synthesizes estradiol from pregnenolone by cytochromes P45017alpha and P450 aromatase localized in neurons. Proc. Natl. Acad. Sci. U.S.A. 101, 865–870 10.1073/pnas.2630225100 14694190 PMC321772

[B19] Hughes P. E. Alexi T. Williams C. E. Clark R. G. Gluckman P. D. (1999). Administration of recombinant human Activin-A has powerful neurotrophic effects on select striatal phenotypes in the quinolinic acid lesion model of Huntington's disease. Neuroscience 92, 197–209 10.1016/S0306-4522(98)00724-6 10392842

[B20] Ikegaya Y. Saito H. Torii K. Nishiyama N. (1997). Activin selectively abolishes hippocampal long-term potentiation induced by weak tetanic stimulation *in vivo*. Jpn. J. Pharmacol. 75, 87–89 10.1254/jjp.75.87 9334889

[B21] Iki J. Inoue A. Bito H. Okabe S. (2005). Bi-directional regulation of postsynaptic cortactin distribution by BDNF and NMDA receptor activity. Eur. J. Neurosci. 22, 2985–2994 10.1111/j.1460-9568.2005.04510.x 16367765

[B22] Inokuchi K. Kato A. Hiraia K. Hishinuma F. Inoue M. Ozawa F. (1996). Increase in activin beta A mRNA in rat hippocampus during long-term potentiation. FEBS Lett. 382, 48–52 10.1016/0014-5793(96)00135-4 8612762

[B23] Irving A. J. Wallace L. Durakoglugil D. Harvey J. (2006). Leptin enhances NR2B-mediated N-methyl-D-aspartate responses via a mitogen-activated protein kinase-dependent process in cerebellar granule cells. Neuroscience 138, 1137–1148 10.1016/j.neuroscience.2005.11.042 16413128 PMC1613257

[B24] Iwahori Y. Saito H. Torii K. Nishiyama N. (1997). Activin exerts a neurotrophic effect on cultured hippocampal neurons. Brain Res. 760, 52–58 10.1016/S0006-8993(97)00275-8 9237517

[B25] Kawato S. Hojo Y. Kimoto T. (2002). Histological and metabolism analysis of P450 expression in the brain. Methods Enzymol. 357, 241–249 10.1016/S0076-6879(02)57682-5 12424914

[B26] Kimoto T. Tsurugizawa T. Ohta Y. Makino J. Tamura H. Hojo Y. (2001). Neurosteroid synthesis by cytochrome p450-containing systems localized in the rat brain hippocampal neurons: N-methyl-D-aspartate and calcium-dependent synthesis. Endocrinology 142, 3578–3589 10.1210/endo.142.8.8327 11459806

[B27] Kitamura T. Saitoh Y. Takashima N. Murayama A. Niibori Y. Ageta H. (2009). Adult neurogenesis modulates the hippocampus-dependent period of associative fear memory. Cell 139, 814–827 10.1016/j.cell.2009.10.020 19914173

[B28] Kokan-Moore N. P. Bolender D. L. Lough J. (1991). Secretion of inhibin beta A by endoderm cultured from early embryonic chicken. Dev. Biol. 146, 242–245 10.1016/0012-1606(91)90464-E 2060706

[B29] Koyano S. Fukui A. Uchida S. Yamada K. Asashima M. Sakuragawa N. (2002). Synthesis and release of activin and noggin by cultured human amniotic epithelial cells. Dev. Growth Differ. 44, 103–112 10.1046/j.1440-169x.2002.00626.x 11940097

[B30] Kramar E. A. Chen L. Y. Brandon N. J. Rex C. S. Liu F. Gall C. M. (2009). Cytoskeletal changes underlie estrogen's acute effects on synaptic transmission and plasticity. J. Neurosci. 29, 12982–12993 10.1523/JNEUROSCI.3059-09.2009 19828812 PMC2806054

[B31] Kramar E. A. Lin B. Lin C. Y. Arai A. C. Gall C. M. Lynch G. (2004). A novel mechanism for the facilitation of theta-induced long-term potentiation by brain-derived neurotrophic factor. J. Neurosci. 24, 5151–5161 10.1523/JNEUROSCI.0800-04.2004 15175384 PMC6729196

[B32] Kurisaki A. Inoue I. Kurisaki K. Yamakawa N. Tsuchida K. Sugino H. (2008). Activin induces long-lasting N-methyl-D-aspartate receptor activation via scaffolding PDZ protein activin receptor interacting protein 1. Neuroscience 151, 1225–1235 10.1016/j.neuroscience.2007.12.012 18201830

[B33] Lai M. Gluckman P. Dragunow M. Hughes P. E. (1997). Focal brain injury increases activin betaA mRNA expression in hippocampal neurons. Neuroreport 8, 2691–2694 10.1097/00001756-199708180-00011 9295102

[B34] Lai M. Sirimanne E. Williams C. E. Gluckman P. D. (1996). Sequential patterns of inhibin subunit gene expression following hypoxic-ischemic injury in the rat brain. Neuroscience 70, 1013–1024 10.1016/0306-4522(95)00413-0 8848164

[B35] Lee M. K. Pardoux C. Hall M. C. Lee P. S. Warburton D. Qing J. (2007). TGF-beta activates Erk MAP kinase signalling through direct phosphorylation of ShcA. EMBO J. 26, 3957–3967 10.1038/sj.emboj.7601818 17673906 PMC1994119

[B36] Lee S. J. Escobedo-Lozoya Y. Szatmari E. M. Yasuda R. (2009). Activation of CaMKII in single dendritic spines during long-term potentiation. Nature 458, 299–304 10.1038/nature07842 19295602 PMC2719773

[B37] Ling N. Ying S. Y. Ueno N. Shimasaki S. Esch F. Hotta M. (1986). A homodimer of the beta-subunits of inhibin A stimulates the secretion of pituitary follicle stimulating hormone. Biochem. Biophys. Res. Commun. 138, 1129–1137 10.1016/S0006-291X(86)80400-4 3092817

[B38] Macqueen G. M. Campbell S. McEwen B. S. Macdonald K. Amano S. Joffe R. T. (2003). Course of illness, hippocampal function, and hippocampal volume in major depression. Proc. Natl. Acad. Sci. U.S.A. 100, 1387–1392 10.1073/pnas.0337481100 12552118 PMC298782

[B39] Mukai H. Hatanaka Y. Mitsuhashi K. Hojo Y. Komatsuzaki Y. Sato R. (2011). Automated analysis of spines from confocal laser microscopy images: application to the discrimination of androgen and estrogen effects on spinogenesis. Cereb. Cortex 21, 2704–2711 10.1093/cercor/bhr059 21527787 PMC3209797

[B40] Mukai H. Tsurugizawa T. Murakami G. Kominami S. Ishii H. Ogiue-Ikeda M. (2007). Rapid modulation of long-term depression and spinogenesis via synaptic estrogen receptors in hippocampal principal neurons. J. Neurochem. 100, 950–967 10.1111/j.1471-4159.2006.04264.x 17266735

[B41] Muller M. R. Zheng F. Werner S. Alzheimer C. (2006). Transgenic mice expressing dominant-negative activin receptor IB in forebrain neurons reveal novel functions of activin at glutamatergic synapses. J. Biol. Chem. 281, 29076–29084 10.1074/jbc.M604959200 16885157

[B42] Niki I. Okazaki K. Saitoh M. Niki A. Niki H. Tamagawa T. (1993). Presence and possible involvement of Ca/calmodulin-dependent protein kinases in insulin release from the rat pancreatic beta cell. Biochem. Biophys. Res. Commun. 191, 255–261 10.1006/bbrc.1993.1210 8383489

[B43] Ooishi Y. Kawato S. Hojo Y. Hatanaka Y. Higo S. Murakami G. (2012a). Modulation of synaptic plasticity in the hippocampus by hippocampus-derived estrogen and androgen. J. Steroid Biochem. Mol. Biol. 131, 37–51 10.1016/j.jsbmb.2011.10.004 22075082

[B44] Ooishi Y. Mukai H. Hojo Y. Murakami G. Hasegawa Y. Shindo T. (2012b). Estradiol rapidly rescues synaptic transmission from corticosterone-induced suppression via synaptic/extranuclear steroid receptors in the hippocampus. Cereb. Cortex 22, 926–936 10.1093/cercor/bhr164 21725036

[B45] Pangas S. A. Woodruff T. K. (2000). Activin signal transduction pathways. Trends Endocrinol. Metab. 11, 309–314 10.1016/S1043-2760(00)00294-0 10996525

[B46] Pi H. J. Lisman J. E. (2008). Coupled phosphatase and kinase switches produce the tristability required for long-term potentiation and long-term depression. J. Neurosci. 28, 13132–13138 10.1523/JNEUROSCI.2348-08.2008 19052204 PMC2620235

[B47] Roberson E. D. English J. D. Adams J. P. Selcher J. C. Kondratick C. Sweatt J. D. (1999). The mitogen-activated protein kinase cascade couples PKA and PKC to cAMP response element binding protein phosphorylation in area CA1 of hippocampus. J. Neurosci. 19, 4337–4348 10341237 10.1523/JNEUROSCI.19-11-04337.1999 PMC6782591

[B48] Shoji-Kasai Y. Ageta H. Hasegawa Y. Tsuchida K. Sugino H. Inokuchi K. (2007). Activin increases the number of synaptic contacts and the length of dendritic spine necks by modulating spinal actin dynamics. J. Cell. Sci. 120, 3830–3837 10.1242/jcs.012450 17940062

[B49] Sumi M. Kiuchi K. Ishikawa T. Ishii A. Hagiwara M. Nagatsu T. (1991). The newly synthesized selective Ca2+/calmodulin dependent protein kinase II inhibitor KN-93 reduces dopamine contents in PC12h cells. Biochem. Biophys. Res. Commun. 181, 968–975 10.1016/0006-291X(91)92031-E 1662507

[B50] Ten Dijke P. Miyazono K. Heldin C. H. (2000). Signaling inputs converge on nuclear effectors in TGF-beta signaling. Trends Biochem. Sci. 25, 64–70 10.1016/S0968-0004(99)01519-4 10664585

[B51] Tretter Y. P. Hertel M. Munz B. Ten Bruggencate G. Werner S. Alzheimer C. (2000). Induction of activin A is essential for the neuroprotective action of basic fibroblast growth factor *in vivo*. Nat. Med. 6, 812–815 10.1038/77548 10888932

[B52] Tretter Y. P. Munz B. Hubner G. Ten Bruggencate G. Werner S. Alzheimer C. (1996). Strong induction of activin expression after hippocampal lesion. Neuroreport 7, 1819–1823 10.1097/00001756-199607290-00026 8905672

[B53] Vale W. Vaughan J. Jolley D. Yamamoto G. Bruhn T. Seifert H. (1986). Assay of growth hormone-releasing factor. Methods Enzymol. 124, 389–401 10.1016/0076-6879(86)24030-6 3086662

[B54] Vlahos C. J. Matter W. F. Hui K. Y. Brown R. F. (1994). A specific inhibitor of phosphatidylinositol 3-kinase, 2-(4-morpholinyl)-8-phenyl-4H-1-benzopyran-4-one (LY294002). J. Biol. Chem. 269, 5241–5248 8106507

[B55] Weed S. A. Du Y. Parsons J. T. (1998). Translocation of cortactin to the cell periphery is mediated by the small GTPase Rac1. J. Cell. Sci. 111(Pt 16), 2433–2443 9683637 10.1242/jcs.111.16.2433

[B56] Werner S. Alzheimer C. (2006). Roles of activin in tissue repair, fibrosis, and inflammatory disease. Cytokine Growth Factor Rev. 17, 157–171 10.1016/j.cytogfr.2006.01.001 16481210

[B57] Wiederrecht G. Lam E. Hung S. Martin M. Sigal N. (1993). The mechanism of action of FK-506 and cyclosporin A. Ann. N.Y. Acad. Sci. 696, 9–19 10.1111/j.1749-6632.1993.tb17137.x 7509138

[B58] Wu D. D. Lai M. Hughes P. E. Sirimanne E. Gluckman P. D. Williams C. E. (1999). Expression of the activin axis and neuronal rescue effects of recombinant activin A following hypoxic-ischemic brain injury in the infant rat. Brain Res. 835, 369–378 10.1016/S0006-8993(99)01638-8 10415398

[B59] Yuste R. Bonhoeffer T. (2001). Morphological changes in dendritic spines associated with long-term synaptic plasticity. Annu. Rev. Neurosci. 24, 1071–1089 10.1146/annurev.neuro.24.1.1071 11520928

