# Supplementary figures and images for: Acute modulation of synaptic plasticity of pyramidal neurons by activin in adult hippocampus
Source: Front Neural Circuits. 2014 Jun 2;8:56. doi: 10.3389/fncir.2014.00056 (PMC4040441; doi:10.3389/fncir.2014.00056)

**Fig. S1**

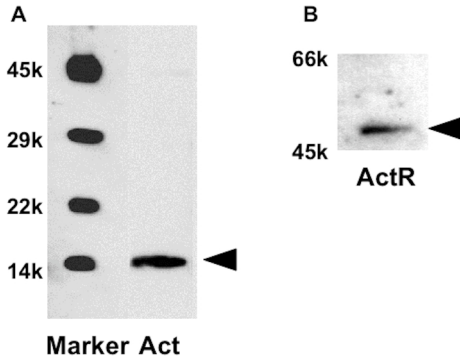

Supplement: Figure S1 — (A) Western blot of activin receptor and activin. ActR, activin receptor. (B) Act, activin; Marker, protein marker. Anti-activin antiserum recognized the main band of 13 kDa activin protein. [file Presentation1.PDF]

Fig 1 A4 absorption control of activin IgG, CA1

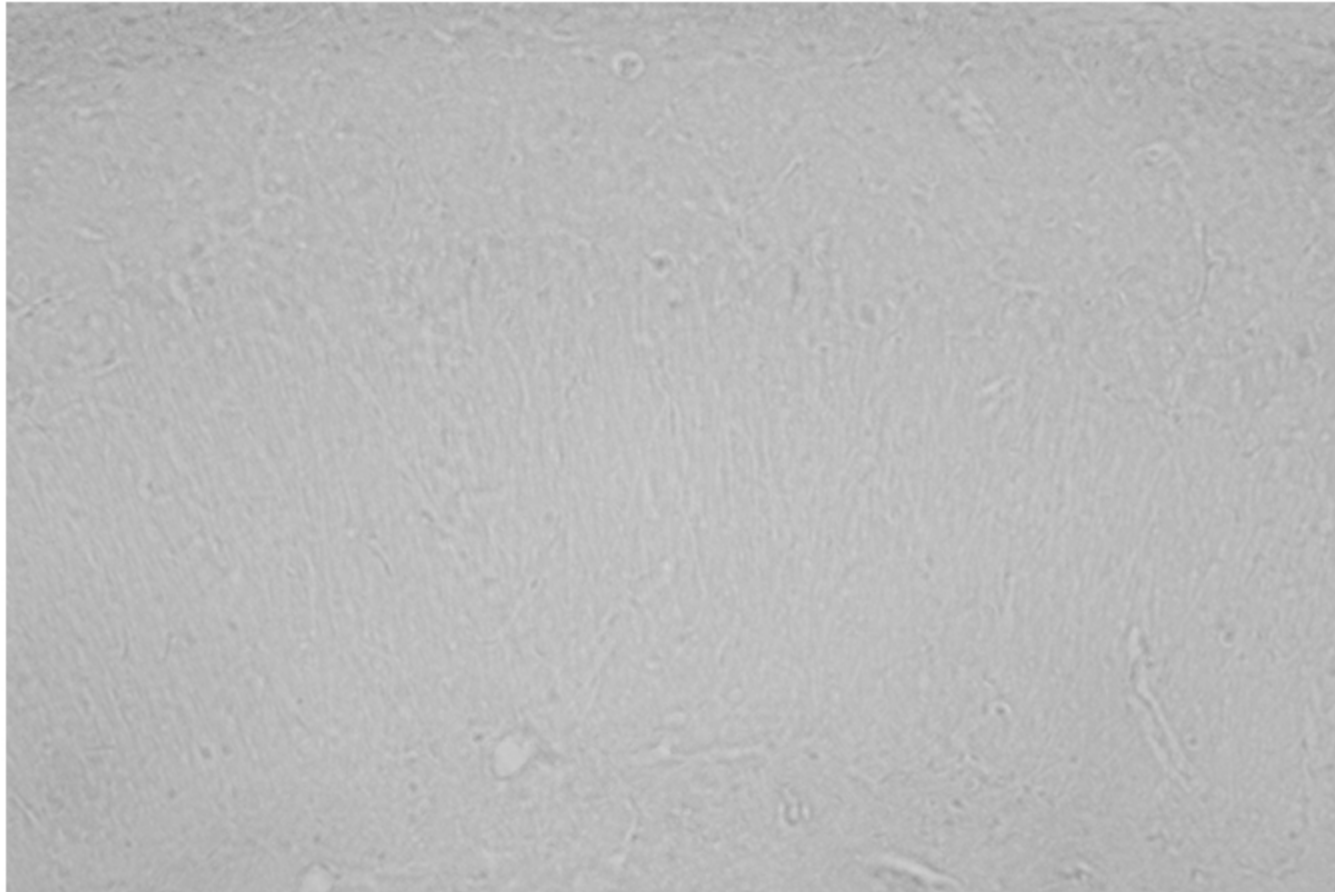

Supplement: Supplementary file 2 [file Presentation2.PDF]

Fig 2A4 absorption control of activin receptor IgG, CA1

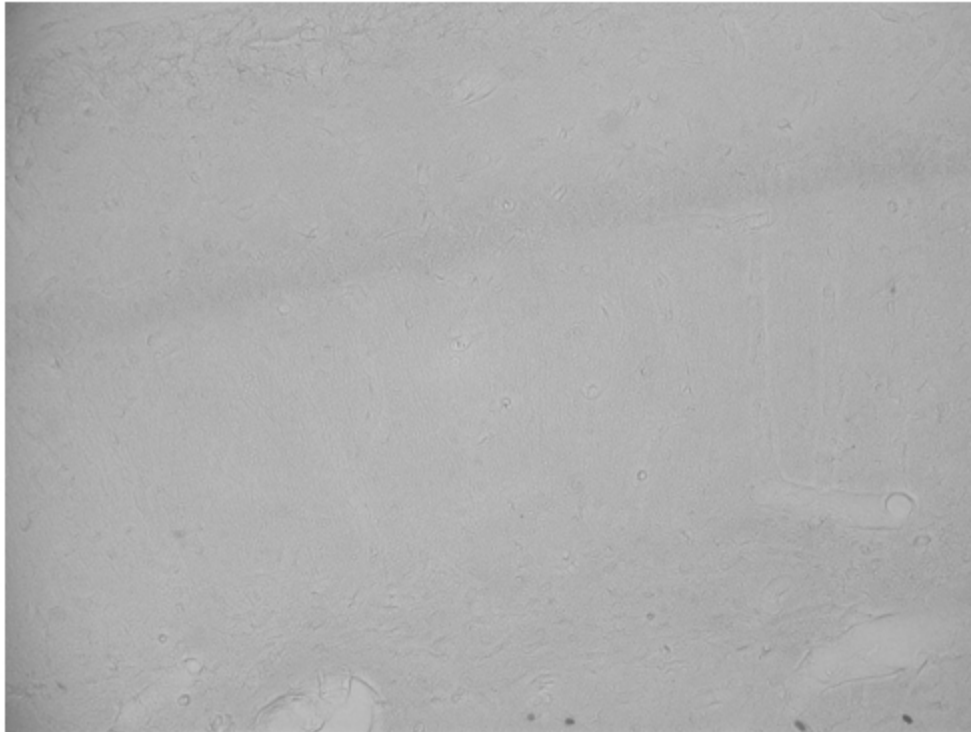

Supplement: Supplementary file 3 [file Presentation3.PDF]
